# Exploring Wound-Healing Genomic Machinery with a Network-Based Approach

**DOI:** 10.3390/ph10020055

**Published:** 2017-06-21

**Authors:** Francesca Vitali, Simone Marini, Martina Balli, Hanne Grosemans, Maurilio Sampaolesi, Yves A. Lussier, Maria Gabriella Cusella De Angelis, Riccardo Bellazzi

**Affiliations:** 1 Center for Biomedical Informatics and Biostatistics, The University of Arizona Health Sciences, Tucson, AZ 85721, USA; francescavitali@email.arizona.edu (F.V.); yves@email.arizona.edu (Y.A.L.); 2 BIO5 Institute Center for Biomedical Informatics and Biostatistics, The University of Arizona, Tucson, AZ 85721, USA; 3 Department of Medicine, The University of Arizona, Tucson, AZ 85721, USA; 4 Department of Electrical, Computer and Biomedical Engineering, University of Pavia, Pavia 27100, Italy; riccardo.bellazzi@unipv.it; 5 Centre for Health Technologies, University of Pavia, Pavia 27100, Italy; 6 Department of Development and Regeneration, Laboratory of Translational Cardiomyology, KULeuven, 3000 Leuven, Belgium; martina.balli01@universitadipavia.it (M.B.); hanne.grosemans@kuleuven.be (H.G.); maurilio.sampaolesi@kuleuven.be (M.S.); 7 Department of Public Health, Experimental and Forensic Medicine, Institute of Human Anatomy, University of Pavia, Pavia 27100, Italy; cusella@unipv.it; 8 Istituti Clinici Scientifici Maugeri, Pavia 27100, Italy

**Keywords:** network pharmacology, gene prioritization, wound healing

## Abstract

The molecular mechanisms underlying tissue regeneration and wound healing are still poorly understood despite their importance. In this paper we develop a bioinformatics approach, combining biology and network theory to drive experiments for better understanding the genetic underpinnings of wound healing mechanisms and for selecting potential drug targets. We start by selecting literature-relevant genes in murine wound healing, and inferring from them a Protein-Protein Interaction (PPI) network. Then, we analyze the network to rank wound healing-related genes according to their topological properties. Lastly, we perform a procedure for in-silico simulation of a treatment action in a biological pathway. The findings obtained by applying the developed pipeline, including gene expression analysis, confirms how a network-based bioinformatics method is able to prioritize candidate genes for in vitro analysis, thus speeding up the understanding of molecular mechanisms and supporting the discovery of potential drug targets.

## 1. Introduction

In recent years, the process of tissue-regeneration has been widely studied to discover treatments for controlling the progression of lesions and for restoring diseased or injured tissue functions. Understanding how damaged, defective (e.g., cancer [1]), or lost tissues can be regenerated is one of the major challenges in biomedical research and in regenerative medicine. The wound healing process plays a key role in tissue regeneration; therefore, a better understanding of the molecules involved in this process, and how they interact, provides new insights into the development of drug treatments. 

The healing process is dependent on interactions between many cell types, extracellular matrix, growth factors, and mediators in a specific temporal phase [2]. Schematically, the healing process can be divided into three different phases: inflammation, proliferation, and remodeling [3]. The time span depends on the agent that affects the wound, the therapy chosen, and the control of the healing environment. In order to successfully heal wounds, all these phases must occur in the proper sequence and time frame. During the healing process some factors could bring delays and slow down the achievement of tissue homeostasis. These factors could include infection, age, sex hormones, stress, diabetes, obesity, medications, alcoholism, smoking, nutrition, and sterile environment [4].

The wound healing underlying genetic mechanisms have been only partially delineated [2,5]. Despite this, several medicine regenerative therapies are currently used to enhance wound healing, such as autologous or allogenic cells, scaffold fabrication, 3D bioprinting, and biomaterials [6]. Among them, our attention is currently devoted to Rigenera^®^ (Human brain wave srl, Turin, Italy), a new autograft protocol utilizing a CE (the abbreviation of the French phrase “Conformité Européene” which literally means “European Conformity”.)-certified medical portable device. Rigenera^®^ produces autologous micro-grafts enriched with progenitor cells, which maintain high cell viability due to its high regenerative potential, allowing the repair of damaged tissues [7]. Moreover, in the Rigenera^®^ protocol, the donor and acceptor are the same individual, preventing possible complications with respect to conventional implants of no autologous micro-grafts [8]. Despite its widespread use in clinical practice [7,8,9,10,11] and its efficacy in boosting tissue regeneration, the genetic and molecular processes regulated by Rigenera^®^ are still unclear. In particular, the mechanism of Rigenera^®^ related to gene expression is still unknown, and further in vitro analysis is required. Current clinical studies based on the Rigenera^®^ protocol attribute the effects of Rigenera^®^ to the presence of mesenchymal stem cells, already analyzed by fluorescence-activated cell sorting (FACS) analyses [7,8,9,11].

In this context, we designed a computational system biology method to unveil biological networks and pathways significantly associated with tissue regeneration, wound healing, and Rigenera^®^. In particular, we applied a network-based strategy: the process under study is represented as a graph, with nodes corresponding to molecular entities of interest (e.g., proteins, genes), and edges to their interactions (e.g., physical/functional interactions). Networks are not only useful tools for integrating different knowledge sources that provide a complete and intuitive representation of the system, but they can also be exploited to extract and predict hidden knowledge, such as biotargets and regulators. Moreover, networks can in-silico simulate qualitative responses to external stimuli (e.g., drug/treatments) under different conditions [12,13,14]. 

Our approach begins with the construction of a Protein-Protein Interaction (PPI) network representing the wound healing process. In this case, network nodes correspond to proteins, while network edges correspond to the interactions between them. Next, network analysis through graph theory identifies relevant candidate nodes as targets for potential treatments. Gene enrichment *p*-values, Boolean network simulations, as well as in vitro experiments on murine cells confirm the involvement of the identified candidate genes in wound healing. In other words, given a poorly understood biological process of interest, we developed and validated an in-silico knowledge-driven pipeline unveiling pivotal molecular elements and molecular drug targets. The implemented procedures are highly customizable and can be used to investigate Rigenera^®^ and wound healing processes as well as deepen our knowledge on other treatments or biological processes. This bioinformatics method is designed to improve the ab initio veracity of hypotheses and to optimize planning of in vitro experiments by researchers for finding new genes, proteins, and pathways involved in different biological processes, diseases, and therapies. 

## 2. Results

Literature analysis and knowledge extracted from public repositories (Section 4.1) led to the selection of a small list of genes related to the wound healing process or to the Rigenera^®^ action. We will refer to this list as the *input genes*. The literature search (performed according to Section 4.1) allowed us to extract a list of seven genes: *Tnf*, *Cxcl2*, *Ccl12*, *Fgf5*, *Wnt5a*, *Col3a1*, *Fosb*, and *Pgk1*; the latter has been included to expand the scope of the network. In fact, besides being a housekeeping gene, *Pgk1* is involved in epidermal regeneration after certain stimuli [15,16]. The input genes and the related literature findings are reported in Table 1.

### 2.1. Assessing the Starting Node Set

**Figure 1:**
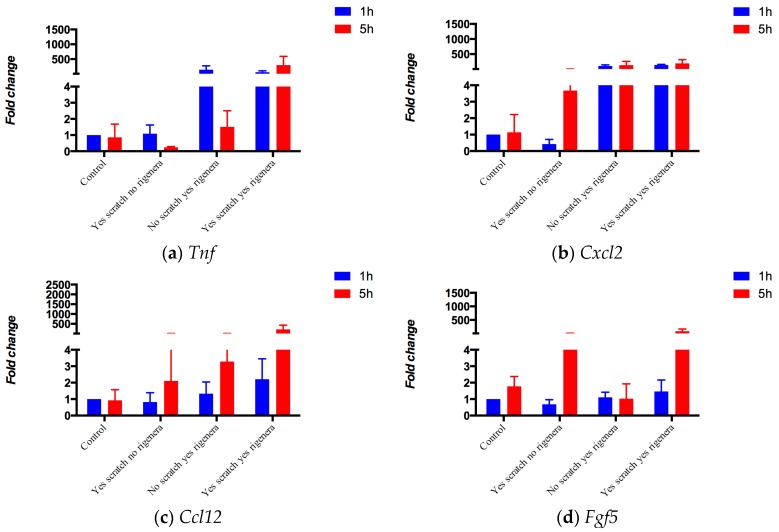

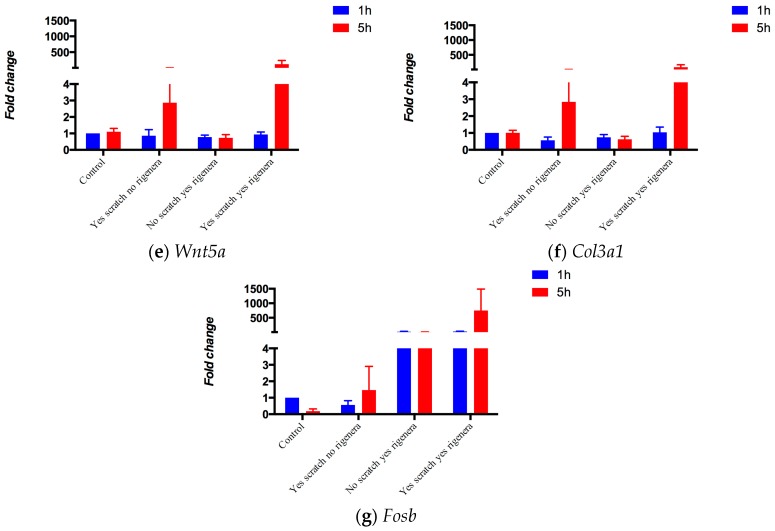
Gene expression after a skin scratch test with and without Rigenera®. (**a**) *Tnf*; (**b**) *Cxcl2*; (**c**) *Ccl12*; (**d**) *Fgf5*; (**e**) *Wnt5a*; (**f**) *Col3a1*; (**g**) *Fosb*.

We verified how the input genes are involved in wound healing and enhanced by Rigenera^®^ via measuring gene expression after a skin scratch test [29] (methods in Section 4.1.1). Figure 1 shows how all the nodes, with the exception of the untested housekeeping *Pgk1*, are stimulated by the Rigenera^®^ action. Moreover, all the genes, except for *Tnf*, are activated after the skin scratch test. These seven genes are important in the three phases of wound healing; inflammation, proliferation, and remodeling. Measured gene expressions are shown in Figure 1. At five hours, the input gene expressions were significantly different between *no Scratch-no Regenera* vs. *yes Scratch-yes Regenera* when taken as a system of seven genes (Z_Stouffer_ = 0.04—Equation (1)), as well as for three individual gene expressions (*Fgf5*, *p*-value = 0.04; *Wnt5a*, *p*-value = 0.03; *Col3a1*, *p*-value = 0.04; *t*-test unequal variance—Section 4.1.1.). As expected at 1 h, the input genes were also significantly dysregulated in three genes (*no Scratch-yes Rigenera*, *Tnf*, *p*-value = 0.03; *no Scratch-yes Rigenera*, *Cxcl2*, *p*-value = 0.02; *no Scratch-yes Rigenera*, *Fosb*, *p*-value = 0.015; *t*-test unequal variance—Section 4.1.1.).

**Figure 2:**
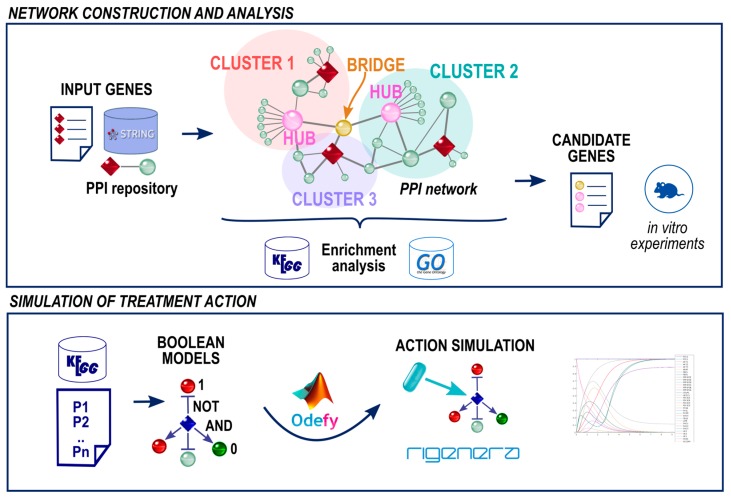
Overview of the proposed computational systems biology method. The network construction is performed through the selection of input genes (shown with red diamonds) involved in the wound healing process. The knowledge about Protein-Protein Interaction (PPI) (STRING repository [30]) is then used to build the network. Next, the topological network analysis selects candidate nodes (i.e., cluster hubs and bridge nodes) and identifies other key genes in the wound healing process. Finally, the simulation of the Rigenera^®^ autologous micro-graft action is performed in the TNF signaling pathway from the Kyoto Encyclopedia of Genes and Genomes (KEGG) [31] through Boolean networks.

We set up a bioinformatics pipeline to infer other genes potentially involved in the wound healing process, a so called *candidate gene set*, based on (a) the starting genes and (b) on the topological knowledge extracted from a protein network. An outline of the presented method is depicted in Figure 2.

### 2.2. A Protein Network for Wound Healing

**Figure 3:**
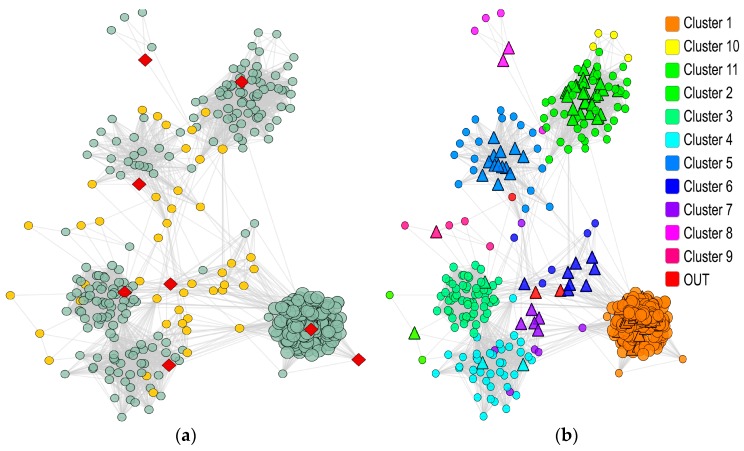
Wound healing PPI network. (**a**) PPI Network. Eight input genes are highlighted in red while the 47 bridge nodes are in yellow; (**b**) Network clustering. The clusters were identified with ClusterONE and are highlighted with different colors. The nodes in the OUT group (see panel (**b**) legend) refer to the nodes that, according to ClusterONE, did not end up in any of the clusters due to their topological properties [33]. The hub nodes of each cluster are shaped with triangles.

We built the wound healing network using the input genes and the STRING repository [30] (Section 4.2). The obtained network, depicted in Figure 3, consists of 446 nodes and 24,757 edges.

To prove the network’s meaningfulness, we performed a KEGG and Gene Ontology (GO) [32] enrichment analysis (Section 4.2.1), finding 62 pathways and 40 GO terms significantly associated with the network (threshold 10^−3^, hypergeometric test), of which 13 pathways and 19 GO terms specifically pertain to wound healing. The complete list is reported in Supplementary Tables S1 and S2.

In the following, we will focus on (a) cluster hubs to study the wound healing machinery with bioinformatics, and (b) bridge nodes to unveil their potential as drug targets with in vitro experiments.

### 2.3. Pivotal Genes in Wound Healing: Hub Nodes of Clusters as the Key Elements in Regulation

Cluster hubs are pivotal nodes in highly connected modules (i.e., clusters) and they have a highly significant number of neighbors. Therefore, these nodes are intrinsically important for understanding the underlying genetic mechanisms.

To identify the hub nodes within a cluster (i.e., cluster hubs), we first partitioned the network with ClusterONE [33]. Of the eleven obtained clusters (Table 2), we retained seven showing a *p*-value < 0.05 (one-sided Mann-Whitney U test). Next, for each cluster, hub nodes were ordered according to their degree (i.e., the number of direct node neighbors), and only the ones ranking beyond the 90th percentile (k > 203.5) were retained (Section 4.3.1) as a candidate gene set (cluster hubs).

Cluster nodes were enriched with KEGG pathways and GO terms. In particular, clusters were characterized by dozens of statistically significant GO terms and KEGG pathways (*p*-value < 0.001, hypergeometric test), as summarized in Table 3 (detailed results are reported in Supplementary Tables S3–S16).

### 2.4. Pivotal Genes in Wound Healing: Bridge Nodes

**Figure 4:**
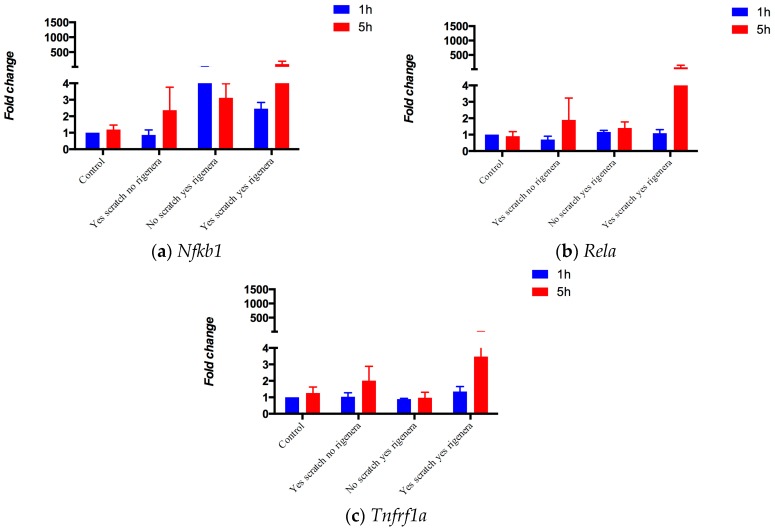
Gene expression after a scratch with and without Rigenera^®^. (**a**) *Nfkb1*; (**b**) *Rela*; (**c**) *Tnfrf1a*.

Bridge nodes are measures of the betweenness of clusters. They are sparsely connected and located among clusters. Identifying bridge proteins is generally done to unveil viable targets for pharmaceuticals [34,35]. By applying the bridging procedure (Section 4.3.2), we identified 45 bridge nodes. Next, we calculated the S_tot_ score (Equation (4)) of each bridge node by considering the wound healing-related KEGG pathways and/or GO terms (Supplementary Tables S17 and S18). The final ranking is reported in Supplementary Table S19, and it represents the estimated likeliness of a bridge node being pivotal in wound healing. Based on the resulting prioritization, we conducted gene expression analysis of the top three scored genes, namely *Nfkb1*, *Rela*, and *Tnfrf1a* (applying the procedure in Section 4.1.1). The experimental results reported in Figure 4 support the findings predicted by the topology analyses. In fact, at five hours, a trend was observed between *no Scratch-no Regenera* vs. *yes Scratch-yes Regenera* for the three output genes (bridge nodes) (Z_Stouffer_ = 0.105—Equation (1); *Tnfrf1a*, *p*-value = 0.086; *Rela*, *p*-value = 0.22; *Nfkb1*, *p*-value = 0.5; *t*-test unequal variance—Section 4.1.1.). 

### 2.5. Simulation of KEGG TNF Signaling Pathway Dynamics

**Figure 5:**
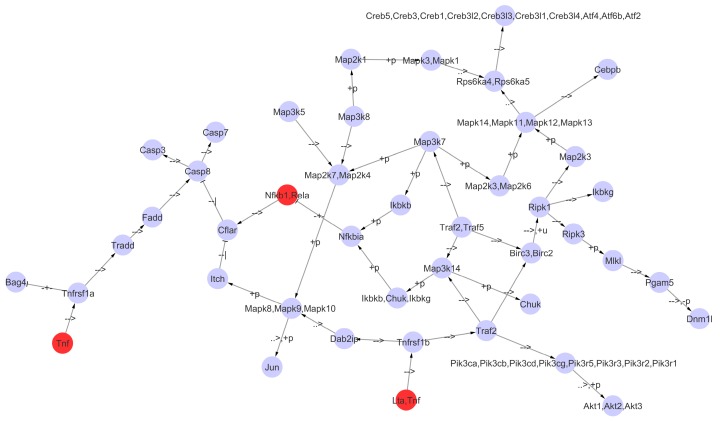
Boolean network of the TNF signaling pathway. Genes whose expression is increased by Rigenera^®^ during the tissue regeneration are highlighted in red.

Following the suggested pipeline, we modeled the KEGG TNF signaling pathway dynamics with a Boolean network (BN) approach (Section 4.4). The resulting network is illustrated in Figure 5 and consists of 44 nodes and 49 edges. 

**Figure 6:**
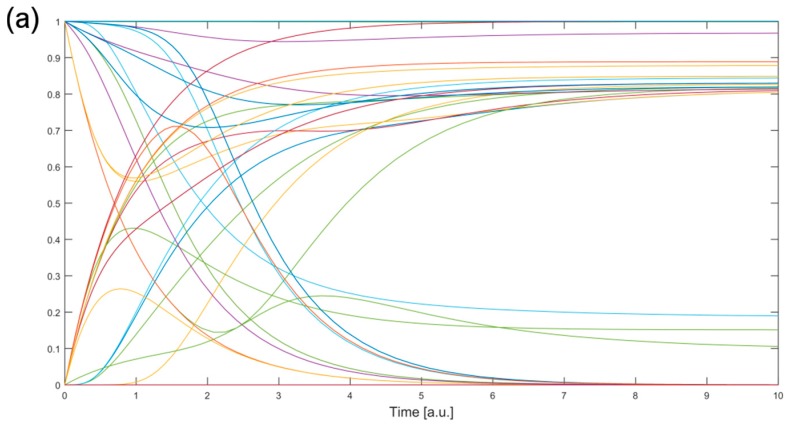

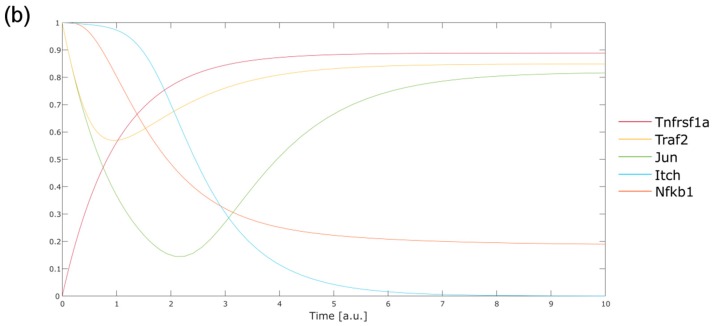
Plots of the TNF pathway node behaviors after the stimulation of Rigenera^®^. The plot has been obtained with *Odefy* and it refers to the output of one Monte Carlo simulation. In detail, Rigenera^®^ autologous micro-graft targets in the pathway have been initialized to their expression values, while other network nodes are randomly set to 0 or 1 (Section 4.4). The plot shows that the values of some genes, initially set to 0, have been increased by the Rigenera^®^ action, and vice versa. Panel (**a**) shows the behaviors of all network nodes; Panel (**b**) shows a subset of such node behaviors, in which *Tnfrsf1a* exhibits an increase, *Itch* a decrease, while Rigenera^®^ seems to not affect the behaviors of *Traf2* and *Jun* (their output values are similar to the initial ones).

Next, we simulated the Rigenera^®^ autologous micro-graft action in the BN by initializing the state (upregulated) of its target genes whose expression is known, i.e., the input nodes (Figure 1) and the candidate gene set (bridges) (Figure 3) overlapping with the TNF pathway. These nodes, *Tnf*, *Rela* and *Nfkb1*, are all set to 1 and are highlighted in red in Figure 5. On the other hand, initial values of the other network nodes are randomly assigned to 0 or 1 (Section 4.4). We next applied *Odefy*, which performs the conversion of BNs into continuous ODEs, with continuous time descriptions, and gives as an output the node behaviors after a certain stimulus (here Rigenera^®^ action). In order to be independent from the random assignment of non-target nodes, we applied *Odefy* with 1000 Monte Carlo simulations, obtaining 1000 behaviors of all network nodes (one example is shown in Figure 6) after the Rigenera^®^ stimulus. The analysis of the results over the simulations allowed us to rank the genes according to the frequency of their state changes. In detail, we looked for the genes that consistently switch their state over the simulations, indicating they have been stimulated by Rigenera^®^. The ranked list is showed in Supplementary Table S20 and the genes in the top 10 are *Bag4*, *Pik3r1*, *Pik3cb*, *Map2k6*, *Map3k7*, *Mapk10*, *Mapk11*, *Pik3ca*, *Map3k14*, and *Atf2*.

The top 10 gene list was analyzed by MouseMine [36]. In Table 4, the GO terms related to wound healing and tissue regeneration are reported.

## 3. Discussion

### 3.1. Automatically Assembling a Gene Network

Our pipeline constructs a PPI network by expanding a list of wound healing-related genes (Table 1). An initial assessment of gene expression levels confirms the input gene list as pertaining to wound healing or to the action of Rigenera^®^ (Figure 1). In fact, all the genes respond positively to the skin scratch test. *Tnf*, *Cxcl2*, *Ccl12*, and *Fosb* are upregulated after the administration of Rigenera^®^ even in the absence of the scratch. When Rigenera^®^ is administered along with the skin scratch test, all the genes show an upregulation, mostly higher than all the other experimental settings, especially after five hours (statistical significance reported in Section 2.1).

After network enrichment, *p*-values of wound healing GO terms and KEGG pathways show how the network well represents the wound recovery process (Supplementary Tables S1 and S2). In the significantly enriched lists, different wound healing-related concepts emerge. Within them there are inflammation (GO:0006954) and angiogenesis (GO:0001525), along with their related terms. The inflammatory response, a well-known phase of the wound healing process, besides being present as a GO term, is underlined by the presence of cytokine-related [37] terms (GO:0016493, GO:0008009, GO:0006935) and pathways (mmu04062, mmu04060, mmu04750). The network is also enriched by the term extracellular matrix (GO:0031012) reflecting the pertinence of its nodes to the tissue regeneration.

The Wnt pathway is activated for cell fate specification, cell proliferation, and cell migration. Wnt is also an important signaling pathway during skin wound healing [38]. Studies show how the expression of Wnt-5a in the wound promotes differences mimicking regeneration, including the development of epithelia-lined cysts in the dermis, hair follicles, and sebaceous glands, without the development of tumors [39]. Notably, an excess of Wnt signaling pathway concepts stands out among the significantly associated terms (GO:0060070, GO:0035567, GO:0090263, GO:0016055, GO:0060071, GO:0042813, GO:0017147) and pathways (mmu04310).

Once the network is assembled, we identified pivotal cluster hubs and bridge nodes. The first are important as actors to understand the wound healing genetic orchestration, and the latter as candidate drug targets.

### 3.2. Unveiling Cluster Hub Nodes

Functional regions, or clusters, allow the segregation of the network in functional modules. These modules are consistently characterized by specific processes (Table 3), reflecting how topologically-differentiated areas of the network are specialized in distinct aspects of wound healing.

- *Cluster 1* gathers chemokine/chemotaxis processes, related to cell migration towards chemical gradients. In particular, chemotaxis plays an important role during the inflammatory phase of healing processes [40].
- *Cluster 2* is labeled by extracellular matrix/skin development terms.
- *Cluster 3* gathers genes involved mainly in DNA regulation of transcription.
- *Cluster 4* gathers regenerative processes and cell growth, being labelled by cancer, pluripotency, Wnt, and mTOR signaling pathways.
- *Cluster 5*, like cluster 2, is characterized by processes of cell adhesion and cell-cell junction formation. This is confirmed by the presence of the Rap1 and Ras signaling pathways, both involved in cell proliferation, survival, growth, migration, differentiation, or cytoskeletal dynamism.
- *Cluster 6* is characterized by inflammation and immune response processes. In fact, it is enriched by terms such as MAPKs, TNF signaling pathway, inflammation regulation, and leukocyte migration. The presence of osteoclast proliferation calcium ion terms is consistent with the involvement of this cluster in new bone formation. Although not directly relevant in wound healing, the fact that bone formation terms were grouped in the same cluster indicates how the clustering technique successfully gathered similar genes in a consistent, meaningful fashion.
- *Cluster 8* is labeled by terms mostly related with Glycine and sugar metabolism. *Pgk1* appears in this cluster, the housekeeping gene involved in glycolysis [15,16].

Within the clusters, cluster hub genes are identified by network theory topological rules. These hubs are genes reflecting the general functions of their respective clusters, and are associated to consistent specific GO terms and KEGG pathways (see Table 3).

### 3.3. Unveiling Bridge Nodes

Bridge nodes have been found to be associated to wound healing-related processes (Supplementary Tables S17 and S18) other than the ones identified for the whole network (Supplementary Tables S1 and S2). This may be due to their special property as individual actors in bridging the different functions related to the various gene clusters. For example, bridge nodes are related to the vascular endothelial growth factor (VEGF) pathway (mmu04370). VEGF plays an important role during the wound healing process, since it is known to be an inducer of cell migration through chemotaxis. It is considered one of the most important factors for its effects on the healing process, including angiogenesis and collagen formation. Different bridge nodes are also related to the apoptotic process (mmu04210). Apoptotic genes play an important role in different physiological processes by removing damaged and potentially dangerous cells. This event is known as apoptosis-induced proliferation or compensatory proliferation and it is important in tissue regeneration [41]. A recent study also shows the involvement of the JAK-STAT signaling pathway (present in the list as mmu04630) in the promotion of compensatory proliferation. This pathway is the principal signaling mechanism for a wide array of cytokines and growth factors [42]. 

Three genes, *Rela*, *Nfkb1*, and *Tnfrsf1a*, are identified as main bridges. Experiments on gene expression show how their response, with or without the Rigenera^®^ stimulus, is dependent on a skin scratch test. *Nfkb1* (Figure 4, panel (a)) responds to the scratch test by increasing its expression level, peaking after five hours. Its expression is also stimulated by Rigenera^®^ in the absence of a skin scratch test. The highest fold change is however reached only when Rigenera^®^ is administered after the scratch test. *Rela* (Figure 4, panel (b)) shows a limited increase in its expression after scratch, or after the sole administration of Rigenera^®^. It shows its highest levels of expression when Rigenera^®^ is administered after a skin scratch test. Finally, the expression of *Tnfrsf1a* (Figure 4, panel (c)) does not seem to be activated by the scratch test; however, it seems to react to the administration of Rigenera^®^ after five hours, without or with the scratch tests. The analysis of the biological results, confirmed that these genes tend to be related to the action of Rigenera^®^ on a skin scratch (Section 2.4). The biological experiments with *n* = 3 in four groups (*no Scratch-no Rigenera^®^*, *no Scratch-yes Rigenera^®^*, *yes Scratch-no Rigenera^®^*, *yes Scratch-yes Rigenera^®^*) were underpowered to evaluate all combinations and control for statistical multiplicity. Therefore, our method suggests three targets, among all possible candidates, for drug discovery or repositioning that may be potentially involved in the wound healing process.

### 3.4. A Boolean Network to Study the TNF Signaling Pathway

Finally, with the modeling of the TNF signaling pathway into a Boolean network we demonstrate the possibility of simulating the action of a treatment (Rigenera^®^) in order to provide qualitative behavior of the genes in a biological pathway. The model pathway TNF has been chosen because of its involvement in chemokine and cytokine pathways and its consequent role in the inflammation phase of wound healing [17]. The analysis of the results obtained by applying *Odefy* enabled the identification of a ranked list of the pathway genes based on the effect of the simulated Rigenera^®^ action. The top 10 genes significantly altered by the Rigenera^®^ action simulation are *Bag4*, *Pik3r1*, *Pik3cb*, *Map2k6*, *Map3k7*, *Mapk10*, *Mapk11*, *Pik3ca*, *Map3k14*, and *Atf2*. Their key role in wound healing was then confirmed by the GO terms related to these genes (Table 4). The p38 MAPKs play a crucial role during the inflammatory phase and they are already known to be involved in tissue regeneration [43]. Moreover, the gene *Map2k6* is the major MAPK11 activator in response to the presence of cytokines, such as TNF-α and IL-1. The Activating transcription factor 2 (*Atf2*) is a member of the leucine zipper family of DNA-binding proteins. *Atf2* is able to respond to stress and may influence cell proliferation, inflammation, apoptosis, oncogenesis, neurological development and function, and skeletal remodeling [44]. The activation of *Atf2* complexes increases the transcription of genes involved in inflammation such as cell adhesion molecules and cytokines, which are important for the recruitment of leukocytes to the site of injury. Finally, for the genes *Pik3r1*, *Pik3cb*, and *Pik3ca*, we found evidence in the literature (i.e., [45]) in addition to the GO terms about their relation to the wound healing process and tissue regeneration. Future studies should include conventional autologous micro-graft controls in order to identify Rigenera^®^-specific networks, which are currently confounded with micro-graft action.

### 3.5. Summary

Our method correctly identifies *Rela*, *Nfkb1*, and *Tnfrsf1a* as drug targets in the wound healing process. It shows how network theories can successfully harvest biological knowledge applied to physiological processes, such as wound healing, and can unveil: (a) candidate targets for drugs/treatments; (b) pivotal genes for the process machinery; and (c) recommendations for in vitro experiments; finally, it simulates the action of a treatment in biological pathways to predict gene responses.

## 4. Materials and Methods

We developed a bioinformatics pipeline (Figure 2) consisting of four different steps: (1) Selection of the *input genes*; (2) Network construction; (3) Identification of *candidate genes (cluster hubs and bridges)*; (3) Gene expression in vitro experiments; and (4) Simulation of a treatment action. The developed pipeline has been implemented in Python 2.7 and Matlab and it is available upon request from the authors. All network figures were created with Cytoscape [46].

### 4.1. Selection of the Input Gene Set

The first step of the proposed approach includes the selection of a set of genes involved in the process under study; in this case, wound healing and, consequently, to Rigenera^®^. The gene list is generated from the literature and public repositories. To this end we looked into PubMed [47], GenBank [48], Uniprot [49], and KEGG [31], and we manually retrieved a gene list on which the network will be constructed, reported in Table 1. We obtained peer-reviewed studies by performing an automated search in PubMed by using combinations of keywords such as “wound-healing”, “gene expression”, “mice”, and “mouse”. This search yielded hundreds of publications; however, we retained only the studies whose findings were experimentally validated. Furthermore, we restricted the search to the experiments where normal vs. scratched tissue or similar approaches were applied (e.g., not considering diabetic mice or experiments involving other treatments). Then relevant studies were selected by first screening titles and abstracts, and then by analyzing full texts and experiment results. In addition, we selected as candidates the genes present on the Mouse Wound Healing RT^2^ Profiler™ PCR Array (Qiagen, Valencia, CA, USA [20]). The genes identified with this procedure were then used to query public repositories to verify their relation to wound-healing. This allowed the selection of a restricted pool of genes on which to perform in vitro experiments to verify their association with Rigenera^®^ and wound healing. In this way, we can assess the methodology as a proof of concept to investigate even poorly understood/studied biological processes and treatments (i.e., processes where only a handful of genes is known to be influential). A detailed description of the method to select the input genes is provided in Supplementary Method S1.

#### 4.1.1. Gene Expression in vitro Experiments

In order to assess if the gene set identified is related to the wound healing process, we performed gene expression assays for the selected genes. 

All in vitro experiments were performed using murine cells, which were cultured from the murine model C57BL/6. By performing a scratch assay, an in vitro damage was created according to the skin scratch test standard practice [50]. Micro-grafts obtained by using the Rigenera^®^ protocol have been added autologously to this model. Cells were cultured in DMEM supplemented with 10% FBS and treated for 1 and 5 h at 37 °C in 5% of CO_2_. 

Gene expression analysis was performed with qRT-PCR on the input genes. For this analysis, total RNA was extracted using the PureLink RNA Mini Kit (Invitrogen) and treated with the DNA-free Kit (Invitrogen). 250 ng RNA was reverse transcribed into cDNA with the Superscript III Reverse Transcriptase First-Strand Synthesis SuperMix (Invitrogen). Quantitative real-time PCR was performed with the Platinum SYBRGreen qPCR SuperMix-UDG (Invitrogen). GraphPad Prism 6 was used to graph the results of the biological replicates ± SEM. The ethical approval code is P056/2017. To assess the statistical significance of the results, we performed the *t*-test controlling for distinct variances to take into account the heteroscedasticity of the results [51]. Once it was assessed that the housekeeping expression values (log2 transformed) of the samples have a similar distribution across the different conditions (i.e., *no Scratch-no Rigenera* vs. *yes Scratch-yes Rigenera*), we could directly perform the *t*-test assuming unequal variance on log2 transformed expressions of the tested genes. Next, on the obtained *p*-values we performed an analysis of the biological validation results as a coordinated gene set/systems/network-level signal. In order to detect a coordinated systems/network-level among input genes, we first summarized the *p*-values of the input gene expression using the Stouffer Z-transform, a meta-analysis [52,53,54] that produces a joint *p*-value for the inputs (Equation (1)). The Z-transform test converts the *t*-test *p*-values, *Px*, for each of *n* transcripts of the gene set into a standard normal curve (*Zx*). The sum of these *Zx*’s, divided by the square root of the number of tests, *n*, has a standard normal [54].

(1) $$Z_{\mathrm{Stouffer}}\;=\frac{\sum_{x=1}^{n}Z_{n}}{\sqrt{n}}$$

### 4.2. Protein-Protein Interaction Network Construction

The PPI network related to the wound healing is derived by integrating the knowledge on PPI from the STRING repository [30]. STRING allows the extraction of PPIs derived from different evidences, i.e., Neighborhoods, Gene Fusion events, Co-occurrence events, Co-expression data, Experimental data, Database information, and Text-mining associations. All these sources of evidence are benchmarked and calibrated against previous knowledge. Through this procedure (detailed in [55]), STRING assigns a *confidence score* to each known or predicted association. This score varies between 0 to 1 and it assesses the quality of the retrieved association based on the related type of evidence (low confidence: scores <0.4, medium: 0.4 to 0.7, and high: >0.7) [55]. In our approach, network nodes correspond to proteins and two proteins are connected by an edge if an interaction between them is found in STRING. Therefore, the entire set of network nodes is determined by considering the input gene set and all its neighbors in STRING (depicted with light green in Figure 2 and Figure 3). The connectivity pattern among nodes is defined according to the STRING interactions and takes into account the *confidence score*. In order to restrict the edge set to the most reliable STRING associations, we introduced two constraints: (i) PPIs need to be derived from Experimental and Database evidence (considered more reliable with respect to, for example, text-mining evidence); and (ii) the STRING *confidence score* has to be higher than 0.7 (high-confidence association). *Confidence scores* have been also integrated into the network model as weights on the network edges.

#### 4.2.1. Enrichment analyses

To assess the meaningful representation of the network and biologically interpret the network genes, we conducted KEGG [56] and Gene Ontology (GO) [32] enrichment analysis of the network nodes. We detected the enrichment on the basis of the hypergeometric test and ranked KEGG pathways and GO terms according to the obtained *p*-values corrected with a false discovery rate (FDR). Statistically significant processes and terms are finally selected by considering only *p*-values < 0.001. 

The resulting significant pathways are then compared to reference lists of KEGG pathways and GO terms known to be related to wound healing. To this end, we manually selected 15 KEGG pathways and 47 GO terms based on their relation with wound healing (Supplementary Tables S1 and S2).

### 4.3. Identification of Candidate Genes

The third step of the proposed procedure aims to identify candidate genes, i.e., network nodes that can be suggested as targets for treatments or other genes that can have a key role in the process. In our approach, such genes are selected based on in-silico analysis of the constructed PPI network. Candidate genes are first selected based on their topological role in the network, and then enrichment selects the best candidates for further investigation. 

Since the structure of the PPI networks reflects the biological properties of the system represented [12,13,57,58], to select candidate genes we introduced two constraints based on the topological property of the network. Candidate genes can correspond to hubs of clusters and bridge nodes. 

#### 4.3.1. Cluster Hubs

In biological networks, and in particular in PPI networks, the topological network structure is characterized by modules. Therefore, network nodes can be clustered into groups (clusters) and these clusters reflect specific biological functions and/or biological processes [13,58]. In graph theory, different clustering algorithms can be applied to detect network clusters [59,60,61]. Our approach uses ClusterONE [33] as it takes into account network weights (i.e., STRING confidence scores) and it allows cluster overlapping. This last property is very important in PPI networks since proteins may have multiple functions and therefore the corresponding nodes may belong to more than one cluster. ClusterONE also provides a *p*-value to select significant clusters, computed through a one-sided Mann-Whitney U test performed on the in-weights and out-weights of cluster vertices. A low *p*-value means that the in-weights are significantly larger than out-weights, so it is more likely that the cluster is a valid finding and not the result of random fluctuations. To distinguish significant clusters from insignificant ones, the authors suggest a *p*-value threshold of 0.05 [33].

Once the significant clusters are identified, in order to assess the meaningfulness of each cluster, KEGG and GO enrichment analyses are performed as described for the network as a whole (Section 4.2.1.). Next, we located cluster hub nodes (i.e., nodes having a number of neighbors higher than average) with consideration of the top 10% of the highest degree nodes. This threshold was suggested by previous works [34,35].

#### 4.3.2. Bridge Nodes

Bridging centrality (BR) is a measure used in graph theory that can discriminate bridge nodes, i.e., the nodes that are crucial to dispatch information to the network topological structures. Bridge nodes usually have fewer neighbors than hubs, and are typically located between highly connected regions (i.e., network modules) [62,63,64]. 

The procedure of identifying bridge nodes begins with computing the BR measure for all network nodes. The BR of a node is defined as the product of the betweenness centrality (BC) and the bridging coefficient (BCoeff). BC measures the local properties of the network, while BCoeff measures global ones. The BR of a node *n* was calculated as proposed by Vitali et al. [34,35] following the equation:

BR(n) = RWBC(n) × BCoeff(n)
(2)

where RWBC is the random walk betweenness centrality [65] and the BCoeff is computed as:

(3) $$\mathrm{BCoeff}\;(n)=\frac{D{\left(n\right)}^{-1}}{\sum_{v\in N\left(n\right)}D{\left(v\right)}^{-1}}$$

where *D*(*n*) is the degree of node *n* (i.e., the number of neighbors of n), and *N*(*n*) is the set of neighbors of node *n*.

Bridge nodes in the network are identified as the nodes whose BR values are in the highest percentiles (i.e., >90%) of the bridging centrality, similar to cluster hub nodes [34,35]. Because of their topological properties, the higher the BR of a node, the more information that flows through it. This makes bridging nodes good drug target candidates. 

Once bridge nodes have been selected, a ranking score function is then applied to identify the most significant candidates for further in vitro experiments. For each bridge node *b*, we automatically retrieved its related KEGG biological pathways and GO terms. Next, by counting the number of pathways and terms related to wound healing, we obtained two first scores $S_{KEGG}\left(b\right)$ and $S_{GO}\left(b\right)$. These scores are then rescaled between 0 to 1 and a global score $S_{tot}$ is assigned to each bridge node *b* according to the formula:

(4) $$S_{tot}\left(b\right)=1-\underset{i=KEGG,GO}{\prod }\left(1-S_{i}\left(b\right)\right)$$

Based on the resulting ranked list, it is possible to plan in vitro experiments for the top scored genes.

### 4.4. How Do Genes React to Rigenera^®^ Stimulus? A Simulation of the Kegg Tnf Signaling Pathway

The fourth step of the proposed approach aims at investigating the effect of Rigenera^®^ on wound healing and suggesting other drug target candidates. To this end, we shift from a PPI representation of the process to a more detailed model. PPI networks can, in fact, provide a static view of the process, because the direction of the interaction (e.g., inhibition or activation) is often unknown. Instead, the molecular network underlying a specific process is highly dynamic: molecules, proteins; and genes associate, dissociate, and interact. In a previous work, we showed how signaling pathways from KEGG can be automatically modeled as BNs [34]. In BNs, nodes are genes and each regulatory reaction in a pathway is translated into a logic formula by parsing the KEGG pathway interactions, according to the conversion rules shown in Supplementary Table S21. BNs can be easily converted into Ordinary Differential Equations (ODEs) for simulation purposes. 

The simulation of the Rigenera^®^ effect in the TNF signaling KEGG pathway (mmu04668) modeled as a BN is performed by initializing the input gene set and the bridge node states according to the treatment action. This means that their state is set to 0 or 1 if the drug, respectively, decreases or increases expression. Since only the initial states of these nodes in the pathway are known, following the procedure exposed in [34], we performed 1000 Monte Carlo simulations randomly assigning 0/1 states to the other nodes. Simulations were performed using the MATLAB-toolbox *Odefy* [66]. Finally, the states of all network nodes after the treatment stimulation are analyzed in order to identify other candidate key genes in the wound healing process. To rank gene candidates, we count the number of times that a gene changes its state from its initial value across all the Monte Carlo simulations, i.e., if the initial gene value passes a 0.5 threshold in any direction then the gene is considered switched.

## Figures and Tables

**Table 1:** Input genes set.

Gene Symbol	Uniprot ID	Protein Names	Ref.
*Tnf*	P06804	Tumor necrosis factor (Cachectin) (TNF-alpha) (Tumor necrosis factor ligand superfamily member 2) (TNF-a)	[17,18,19,20]
*Cxcl2*	P10889	C-X-C motif chemokine 2 (Macrophage inflammatory protein 2) (MIP2)	[18,19,21]
*Ccl12*	Q62401	C-C motif chemokine 12 (MCP-1-related chemokine) (Monocyte chemoattractant protein 5) (Monocyte chemotactic protein 5) (MCP-5) (Small-inducible cytokine A12)	[20,22]
*Fgf5*	P15656	Fibroblast growth factor 5 (FGF-5) (Heparin-binding growth factor 5) (HBGF-5)	[19,23]
*Wnt5a*	P22725	Protein Wnt-5a	[20,24,25]
*Col3a1*	P08121	Collagen alpha-1(III) chain	[20,26,27,28]
*Fosb*	P13346	Protein fosB	[22]
*Pgk1*	P09411	Phosphoglycerate kinase 1	[15,16]

**Table 2:** Network clusters identified with ClusterONE.

Cluster	Size	Density	Internal Weight	External Weight	*p*-Value	# of Hubs
1 *	207	0.8654	1.85 × 10^4^	66.97	<2.2204 × 10^−16^ **	45
2 *	66	0.5888	1263	32.96	<2.2204 × 10^−16^ **	20
3 *	58	0.8127	1343	103.4	<2.2204 × 10^−16^ **	8
4 *	42	0.6027	518.9	116.2	<2.2204 × 10^−16^ **	5
5 *	32	0.5541	274.8	23.84	<2.2204 × 10^−16^ **	13
6 *	13	0.7173	55.95	49.28	7.27 × 10^−5^	4
7	11	0.675	37.12	77.5	0.103808	5
8 *	6	0.54	8.1	0.8	0.00150023	2
9	5	0.6669	6.669	7.334	0.0712283	1
10	4	0.9	5.4	14.4	0.997531	0
11	3	0.5987	1.796	4.2	0.5	1

* Significant clusters (*p*-value < 0.05); ** 2.2204 × 10^−16^ is the machine epsilon; *Internal weight* refers to the total weight of the edges contained in the cluster; *External weight* denotes the total weight of the edges that connect the cluster nodes with the rest of the network [33].

**Table 3:** The number of statistically significant GO terms and KEGG pathways per cluster, characterizing terms in the clusters by (1) all significant nodes, and (2) hub nodes.

Cluster	# Significant GO Terms	# Significant KEGG Pathways	Top GO Labels (Net Count)	Top KEGG Pathways
1	15	35	G-protein coupled receptor signaling pathway **chemotaxis** **C-C chemokine receptor activity**	Neuroactive ligand-receptor interaction **Chemokine signaling pathway**
2	23	16	**basement membrane** external side of plasma membrane **extracellular matrix**	ECM-receptor interaction **Focal adhesion** **PI3K-Akt signaling pathway**
3	16	2	positive regulation of transcription negative regulation of transcription regulation of transcription	**Adipocytokine signaling pathway** Thyroid hormone signaling pathway
4	48	10	positive regulation of transcription **canonical Wnt signaling pathway** **Wnt-protein binding**	**Wnt signaling pathway** Breast cancer **mTOR signaling pathway**
5	46	20	**positive regulation of cell proliferation** lung development cell surface	**Rap1 signaling pathway** **Ras signaling pathway** **PI3K-Akt signaling pathway**
6	9	53	regulation of transcription positive regulation of transcription from RNA polymerase II promoter **cellular response to calcium ion**	Osteoclast differentiation **MAPK signaling pathway** **TNF signaling pathway**
8	2	6	phosphoglycerate mutase activity glycolytic process	Glycine Glycolysis/Gluconeogenesis Metabolic pathways

Number of statistically significant GO terms and KEGG pathways per cluster. The three most frequent terms by net count are reported, with terms related to the wound healing process in bold. Details about significant hub GO terms and KEGG pathways are reported in Supplementary Tables S3–S16.

**Table 4:** GO biological processes associated with the top 10 genes stimulated by Rigenera^®^.

Gene	GO Term ID	GO Term Name
*Bag4*	GO:0006915	apoptotic process
	GO:0010763	positive regulation of fibroblast migration
	GO:0030838	positive regulation of actin filament polymerization
	GO:0042981	regulation of apoptotic process
	GO:0045785	positive regulation of cell adhesion
	GO:0051496	positive regulation of stress fiber assembly
	GO:0071364	cellular response to epidermal growth factor stimulus
*Pik3r1*	GO:0001953	negative regulation of cell-matrix adhesion
	GO:0007162	negative regulation of cell adhesion
	GO:0008625	extrinsic apoptotic signaling pathway via death domain receptors
	GO:0043066	negative regulation of apoptotic process
	GO:0030335	positive regulation of cell migration
*Pik3cb*	GO:0001935	endothelial cell proliferation
	GO:0001952	regulation of cell-matrix adhesion
	GO:0007155	cell adhesion
	GO:0009611	response to wounding
	GO:0030168	platelet activation
	GO:0060055	angiogenesis involved in wound healing
*Map2k6*	GO:0043065	positive regulation of apoptotic process
*Map3k7*	GO:0006915	apoptotic process
	GO:0016239	positive regulation of macroautophagy
	GO:1902443	negative regulation of ripoptosome assembly involved in necroptotic process
*Mapk10*	GO:0006468	protein phosphorylation
*Mapk11*	GO:0006468	protein phosphorylation
	GO:0006950	response to stress
	GO:0016310	phosphorylation
*Pik3ca*	GO:2000270	negative regulation of fibroblast apoptotic process
	GO:0016310	phosphorylation
*Map3k14*	GO:0006468	protein phosphorylation
	GO:0006955	immune response
	GO:0016310	phosphorylation
	GO:0030036	actin cytoskeleton organization
*Atf2*	GO:1902110	positive regulation of mitochondrial membrane permeability involved in apoptotic process

## Supplementary Materials

The following are available online at www.mdpi.com/1424-8247/10/2/55/s1. Table S1: KEGG pathways significantly associated to the whole network, Table S2: GO terms significantly associated to the whole network ; Table S3 to Table S16: GO/KEGG enrichment of the clusters; Table S17: KEGG pathways related to the wound healing and to at least one bridge node; Table S18: GO terms related to the wound healing and to at least one bridge node; Table S19: Bridge node score S; Table S20: Ordered list of genes changed over the simulations; Table S21: Table to convert KEGG associations into Boolean rules; and Method S1: Input gene panel setup.
