# Supplementary material for: Exploring Wound-Healing Genomic Machinery with a Network-Based Approach
Source: Pharmaceuticals (Basel). 2017 Jun 21;10(2):55. doi: 10.3390/ph10020055 (PMC5490412; doi:10.3390/ph10020055)
Supplement: Supplementary file 1 [file pharmaceuticals-10-00055-s001.pdf]

pharmaceuticals

Supplementary Material: Exploring wound-healing genomic machinery with a network-based approach

Francesca Vitali, Simone Marini, Martina Balli, Hanne Grosemans, Maurilio Sampaolesi, Yves A. Lussier, Maria Gabriella Cusella De Angelis and Riccardo Bellazzi

Table S1. KEGG pathways significantly associated to the whole network.

KEGG ID	Name	*p*-Value
mmu04920	Adipocytokine signaling pathway	1.21E-06
mmu04933	AGE-RAGE signaling pathway in diabetic complications	6.33E-07
mmu05412	Arrhythmogenic right ventricular cardiomyopathy (ARVC)	1.20E-08
mmu05217	Basal cell carcinoma	2.63E-21
mmu05224	Breast cancer	1.80E-28
**mmu04024***	**cAMP signaling pathway**	**3.68E-10**
mmu05142	Chagas disease (American trypanosomiasis)	7.35E-07
**mmu04062***	**Chemokine signaling pathway**	**2.02E-24**
mmu04725	Cholinergic synapse	0.000418
mmu04713	Circadian entrainment	8.32E-05
mmu05030	Cocaine addiction	3.26E-05
**mmu04060***	**Cytokine-cytokine receptor interaction**	**3.19E-15**
mmu05414	Dilated cardiomyopathy	1.37E-15
mmu04512	ECM-receptor interaction	4.79E-26
mmu01522	Endocrine resistance	2.64E-10
mmu04915	Estrogen signaling pathway	8.32E-05
**mmu04510***	**Focal adhesion**	**2.46E-17**
mmu04727	GABAergic synapse	0.000902
mmu04540	Gap junction	1.53E-05
mmu04971	Gastric acid secretion	0.000156
mmu04724	Glutamatergic synapse	5.36E-05
mmu04912	GnRH signaling pathway	5.99E-06
mmu04390	Hippo signaling pathway	7.15E-09
mmu05166	HTLV-I infection	1.37E-12
mmu05410	Hypertrophic cardiomyopathy (HCM)	2.07E-08
**mmu04750***	**Inflammatory mediator regulation of TRP channels**	**0.00062**
mmu05140	Leishmaniasis	0.000608
mmu04211	Longevity regulating pathway	0.000198
mmu04213	Longevity regulating pathway - multiple species	0.000136
**mmu04010***	**MAPK signaling pathway**	**6.22E-07**
mmu04916	Melanogenesis	1.19E-28
mmu05218	Melanoma	3.33E-08
mmu05032	Morphine addiction	3.80E-05
**mmu04150***	**mTOR signaling pathway**	**4.81E-12**
mmu04080	Neuroactive ligand-receptor interaction	5.00E-24
mmu04621	NOD-like receptor signaling pathway	9.71E-06

mmu04380	Osteoclast differentiation	0.000368
mmu04913	Ovarian steroidogenesis	0.000844
mmu05200	Pathways in cancer	6.20E-40
mmu05133	Pertussis	1.50E-08
mmu04072	Phospholipase D signaling pathway	1.25E-06
**mmu04151***	**PI3K-Akt signaling pathway**	**4.05E-26**
**mmu04611***	**Platelet activation**	**6.23E-08**
mmu04914	Progesterone-mediated oocyte maturation	3.04E-07
mmu04974	Protein digestion and absorption	3.19E-17
mmu05205	Proteoglycans in cancer	7.82E-21
**mmu04015**	**Rap1 signaling pathway**	**5.24E-24**
**mmu04014**	**Ras signaling pathway**	**6.37E-09**
mmu04810	Regulation of actin cytoskeleton	5.73E-14
mmu04923	Regulation of lipolysis in adipocytes	5.48E-09
mmu04723	Retrograde endocannabinoid signaling	1.50E-07
mmu04622	RIG-I-like receptor signaling pathway	6.19E-05
mmu05132	Salmonella infection	0.000274
mmu04550	Signaling pathways regulating pluripotency of stem cells	1.57E-17
mmu05222	Small cell lung cancer	1.32E-05
mmu04071	Sphingolipid signaling pathway	3.04E-07
mmu04742	Taste transduction	4.73E-27
mmu04919	Thyroid hormone signaling pathway	4.15E-07
**mmu04668***	**TNF signaling pathway**	**1.05E-09**
mmu04620	Toll-like receptor signaling pathway	0.000399
mmu05145	Toxoplasmosis	0.000459
**mmu04310***	**Wnt signaling pathway**	**3.57E-21**

* Related to the wound healing in the network.

Table S2. GO terms significantly associated to the whole network.

GO Term	Name	*p*-Value	GO Aspects
GO:0004016	adenylate cyclase activity	9.48E-06	F
GO:0007193	adenylate cyclase-inhibiting G-protein coupled receptor signaling pathway	3.82E-08	P
**GO:0001525***	**angiogenesis**	**7.82E-07**	**P**
**GO:0005604***	**basement membrane**	**6.81E-06**	**C**
**GO:0060070***	**canonical Wnt signaling pathway**	**1.46E-11**	**P**
**GO:0016493***	**C-C chemokine receptor activity**	**2.75E-10**	**F**
GO:0009986	cell surface	5.16E-07	C
GO:0071230	cellular response to amino acid stimulus	6.66E-07	P
**GO:0008009***	**chemokine activity**	**0.000204**	**F**
**GO:0006935***	**chemotaxis**	**2.84E-09**	**P**
GO:0030199	collagen fibril organization	0.000204	P
GO:0005581	collagen trimer	5.94E-11	C
GO:0005587	collagen type IV trimer	6.66E-07	C
GO:0009897	external side of plasma membrane	3.65E-06	C
**GO:0031012***	**extracellular matrix**	**0.000168**	**C**
GO:0005109	frizzled binding	9.89E-07	F
GO:0007186	G-protein coupled receptor signaling pathway	2.52E-08	P
**GO:0006954***	**inflammatory response**	**1E-4**	**P**
GO:0030324	lung development	4.35E-06	P
GO:0016592	mediator complex	6.49E-30	C
GO:0007218	neuropeptide signaling pathway	5.71E-05	P
GO:0004982	N-formyl peptide receptor activity	6.66E-07	F
**GO:0035567***	**non-canonical Wnt signaling pathway**	**0.000171**	**P**
GO:0001601	peptide YY receptor activity	0.000636	F
GO:1904938	planar cell polarity pathway involved in axon guidance	0.000636	P
**GO:0090263***	**positive regulation of canonical Wnt signaling pathway**	**7.63E-09**	**P**
**GO:0008284***	**positive regulation of cell proliferation**	**1.84E-05**	F
**GO:0050679***	**positive regulation of epithelial cell proliferation**	**0.000204**	P
**GO:0043410***	**positive regulation of MAPK cascade**	**0.000557**	**P**
**GO:0001934***	**positive regulation of protein phosphorylation**	**3.21E-05**	**P**
GO:0036342	post-anal tail morphogenesis	8.18E-06	P
GO:0005578	proteinaceous extracellular matrix	1.27E-05	C
GO:0060665	regulation of branching involved in salivary gland morphogenesis by mesenchymal-epithelial signaling	0.000636	P
GO:0035994	response to muscle stretch	0.000636	P
GO:0019827	stem cell population maintenance	0.000204	P
**GO:0072678***	**T cell migration**	**0.000146**	**P**
**GO:0016055***	**Wnt signaling pathway**	**2.17E-06**	**P**

**GO:0060071***	**Wnt signaling pathway, planar cell polarity pathway**	**2.17E-06**	**P**
**GO:0042813***	**Wnt-activated receptor activity**	**9.60E-06**	**F**
**GO:0017147***	**Wnt-protein binding**	**5.11E-12**	**F**
**GO:0050679***	**positive regulation of epithelial cell proliferation**	**0.000204**	**P**
**GO:0043410***	**positive regulation of MAPK cascade**	**0.000557**	**P**
**GO:0001934***	**positive regulation of protein phosphorylation**	**3.21E-05**	**P**
GO:0036342	post-anal tail morphogenesis	8.18E-06	P

* Related to the wound healing in the network; GO aspects: F = Molecular function; P= Biological process and C = Cellular component.

Table S3. Cluster 1 enriched GO terms.

GO_ID	GO_NAME	p-Value	Count Tot	Net Count	Genes
GO:0007193	adenylate cyclase-inhibiting G-protein coupled receptor signaling pathway	2.57E-11	13	9	Ptgdr2, S1pr3, S1pr1, Npy2r, Oprm1, Sstr2, Oprd1, Lpar1, Gnai2
GO:0006935	chemotaxis	4.15E-12	27	12	Ptgdr2, Ccr10, Xcr1, Ccl20, Ccr3, Ccr4, Ccr6, Ccr7, Ccr8, Ccr9, Ccl25, S1pr1
GO:0004982	N-formyl peptide receptor activity	4.90E-09	6	6	Fpr-rs7, Fpr-rs6, Fpr-rs4, Fpr-rs3, Fpr1, Fpr2
GO:0001602	pancreatic polypeptide receptor activity	0.000288	3	3	Npy1r, Npy4r, Npy5r
GO:0001591	dopamine neurotransmitter receptor activity, coupled via Gi/Go	0.000288	3	3	Drd4, Drd2, Drd3
GO:0030595	leukocyte chemotaxis	0.000587	9	4	Ccr1, S1pr1, Cnr2, Cxcr5
GO:0007186	G-protein coupled receptor signaling pathway	1.13E-13	59	17	Fpr-rs7, Rgs7, Fpr1, Adra2c, Gnb1, Adra2a, Rgs18, Drd2, Sstr1, Npy2r, Sstr2, Gng2, Adra2b, Lpar2, Lpar3, Gnat3, Lpar1
GO:0004983	neuropeptide Y receptor activity	0.000983	4	3	Npy2r, Npy1r, Npy5r
GO:0004985	opioid receptor activity	0.000288	3	3	Oprk1, Oprm1, Oprd1
GO:0007218	neuropeptide signaling pathway	6.89E-07	10	6	Nmur2, Nmur1, Sstr2, Sstr1, Gal, Oprd1
GO:0016493	C-C chemokine receptor activity	1.13E-13	12	10	Ccr10, Ccr1, Ccr2, Ccr3, Ccr4, Ccr5, Ccr6, Ccr7, Ccr8, Ccr9
GO:0004016	adenylate cyclase activity	2.19E-07	5	5	Adcy5, Adcy6, Adcy7, Adcy9, Adcy3
GO:0001601	peptide YY receptor activity	3.85E-05	5	4	Npy2r, Npy1r, Npy4r, Npy5r
GO:0008009	chemokine activity	2.57E-06	12	6	Cxcl3, Ppbp, Cxcl13, Cxcl12, Ccl25, Cxcl16
GO:0006171	cAMP biosynthetic process	0.000288	3	3	Adcy6, Adcy9, Adcy3

[1] *Count tot* is the number of genes associated to the GO term; [2] *Net count* is the number of genes associated to the GO term *and* present in the cluster.

Table S4. Cluster 2 enriched KEGG pathways.

KEGG_ID	KEGG_NAME	*p*-Value	Tot Count	Net Count	Genes
mmu04024	cAMP signaling pathway	2.43E-12	199	28	Adcy1, Adcy2, Adcy5, Adcy6, Adora1, Sucnr1, Adcy9, Hcar2, Adcy4, Adcy3, Sstr2, Npy, Chrm2, Gnai1, Gnai2, Gnai3, Adcy8, Sstr5, Npy1r, Drd2, Adcy7, Sstr1, Htr1a, Htr1b, Htr1d, Htr1f, Hcar1, Ptger3
mmu05200	Pathways in cancer	0.000642	395	23	Gnb1, Adcy1, Adcy2, Adcy5, Adcy6, Adcy7, Bdkrb1, Adcy9, Bdkrb2, Lpar2, Adcy4, Adcy3, Cxcl12, Lpar1, Gnai1, Lpar3, Gng2, Gnai2, Gnai3, Cxcr4, Lpar5, Adcy8, Ptger3
mmu04923	Regulation of lipolysis in adipocytes	8.03E-12	57	16	Gnai3, Gnai1, Adcy4, Adcy3, Adcy2, Adcy5, Adcy7, Npy1r, Adcy6, Adora1, Adcy8, Adcy9, Adcy1, Npy, Gnai2, Ptger3
mmu04925	Aldosterone synthesis and secretion	0.000982	86	9	Adcy1, Adcy3, Adcy2, Adcy5, Adcy4, Adcy6, Adcy7, Adcy8, Adcy9
mmu04062	Chemokine signaling pathway	3.15E-39	196	53	Gnb1, Ppbp, Xcr1, Adcy2, Adcy5, Cxcr6, Adcy6, Adcy7, Adcy8, Adcy9, Gng2, Cxcr5, Ccr7, Gm2023, Ccr10, Ccr8, Ccr3, Ccr1l1, Ccr4, Ccl25, Ccr6, Adcy3, Cxcl5, Adcy1, Cxcl2, Cxcl16, Cxcl12, Gnai1, Gnai2, Cxcl9, Ccl28, Gm1987, Cxcl1, Ccr5, Ccl20, Cxcl3, Gnai3, Ccl12, Cxcl10, Arrb2, Cxcr1, Cxcr3, Cxcr4, Cxcr2, Ccr2, Ccr1, Ccr9, Adcy4, Cx3cr1, Ccl6, Ccl5, Cxcl13, Ccl9
mmu04080	Neuroactive ligand-receptor interaction	1.58E-44	285	66	Galr1, Hrh3, Fpr1, Fpr3, Mtnr1b, S1pr2, Aplnr, C5ar1, Agtr2, Adra2b, Galr2, Adora1, Bdkrb1, Bdkrb2, Grm2, Grm3, S1pr5, Chrm4, Oprl1, Drd4, Drd3, Oprd1, Sstr5, Sstr1, Sstr2, Sstr3, S1pr4, S1pr3, Rxfp3, Cnr2, Cnr1, Mchr1, Nmur1, P2ry14, Grm8, Lpar2, Htr5a, Lpar1, Fpr2, Lpar3, P2ry13, Nmur2, Chrm2, Adra2a, C3ar1, Grm6, Grm7, Rxfp4, Npbwr1, Mtnr1a, Adra2c, Oprk1, Oprm1, P2ry4, Npy5r, S1pr1,

					Npy1r, Npy2r, Drd2, Hrh4, Npy4r, Htr1a, Htr1b, Htr1d, Htr1f, Ptger3
mmu04742	Taste transduction	4.61E-41	88	41	Tas2r107, Tas2r109, Tas2r104, Tas2r106, Tas2r114, Tas2r118, Tas2r110, Tas2r113, Tas2r120, Tas2r121, Tas2r123, Tas2r117, Tas2r136, Adcy6, Adcy8, Tas2r139, Tas2r137, Tas2r115, Adcy4, Tas2r119, Gnat3, Tas2r108, Tas2r105, Tas2r102, Tas2r124, Tas2r126, Tas2r125, Tas2r130, Tas2r129, Tas2r131, Tas2r144, Tas2r138, Tas2r135, Tas2r116, Tas2r140, P2ry4, Tas2r103, Htr1a, Htr1b, Htr1d, Htr1f
mmu04071	Sphingolipid signaling pathway	0.000982	124	11	Oprd1, Gnai1, S1pr2, S1pr1, Gnai2, Gnai3, S1pr4, S1pr3, S1pr5, Adora1, Bdkrb2
mmu04725	Cholinergic synapse	1.89E-07	112	16	Gnb1, Gnai1, Chrm2, Adcy1, Adcy3, Adcy2, Adcy5, Gnai3, Adcy4, Adcy6, Adcy7, Adcy8, Adcy9, Gng2, Gnai2, Chrm4
mmu04015	Rap1 signaling pathway	3.28E-06	215	20	Lpar2, Fpr1, Gnai1, Lpar1, Lpar3, Adcy1, Adcy3, Adcy2, Adcy5, Adcy7, Drd2, Gnai3, Adcy4, Adcy6, Rgs14, Lpar5, Adcy9, Cnr1, Adcy8, Gnai2
mmu04723	Retrograde endocannabinoid signaling	2.91E-07	102	15	Gnb1, Cnr1, Adcy4, Adcy3, Adcy2, Adcy5, Gnai3, Gng2, Adcy6, Adcy7, Adcy8, Adcy9, Adcy1, Gnai2, Gnai1
mmu04060	Cytokine-cytokine receptor interaction	3.19E-17	264	38	Ppbp, Xcr1, Cxcr6, Cxcr5, Ccr7, Gm2023, Ccr10, Ccr8, Ccr3, Ccr1l1, Ccr4, Ccr2, Ccr6, Ackr3, Cxcl2, Cxcl16, Cxcl12, Cxcl9, Ccl28, Gm1987, Cxcl1, Ccr5, Ccl20, Cxcl5, Ccl12, Cxcl10, Cxcr1, Cxcr3, Cxcr4, Cxcr2, Ccr1, Ccr9, Ccl25, Cx3cr1, Ccl6, Ccl5, Cxcl13, Ccl9
mmu04724	Glutamatergic synapse	9.50E-10	114	19	Grm8, Gnb1, Gnai1, Adcy1, Adcy3, Adcy2, Adcy5, Gnai3, Gnai2, Adcy4, Adcy6, Adcy7, Adcy8, Adcy9, Grm7, Grm2, Grm3, Gng2, Grm6
mmu04726	Serotonergic synapse	0.00051	132	12	App, Htr5a, Gnb1, Gnai1, Gng2, Gnai2, Adcy5, Gnai3, Htr1a, Htr1b, Htr1d, Htr1f

mmu04727	GABAergic synapse	2.91E-07	88	14	Gnb1, Adcy5, Adcy1, Adcy3, Adcy2, Gnai3, Adcy4, Adcy6, Adcy7, Adcy8, Adcy9, Gng2, Gnai2, Gnai1
mmu05030	Cocaine addiction	0.000114	48	8	Gnai3, Gnai1, Gnai2, Adcy5, Drd2, Pdyn, Grm2, Grm3
mmu04611	Platelet activation	6.93E-05	123	13	Gnai3, Adcy5, Adcy1, Adcy3, Adcy2, P2ry12, Adcy4, Adcy6, Adcy7, Adcy8, Adcy9, Gnai2, Gnai1
mmu04213	Longevity regulating pathway - multiple species	0.00014	64	9	Adcy4, Adcy3, Adcy2, Adcy5, Adcy1, Adcy6, Adcy7, Adcy8, Adcy9
mmu04261	Adrenergic signaling in cardiomyocytes	0.00051	152	13	Gnai3, Gnai1, Adcy1, Adcy3, Adcy2, Adcy5, Agtr2, Adcy4, Adcy6, Adcy7, Adcy8, Adcy9, Gnai2
mmu04971	Gastric acid secretion	3.87E-08	74	14	Gnai3, Gnai1, Adcy1, Adcy3, Adcy2, Adcy5, Gnai2, Sst, Adcy6, Adcy7, Adcy8, Adcy9, Adcy4, Sstr2
mmu04970	Salivary secretion	0.000543	78	9	Adcy1, Adcy3, Adcy2, Adcy5, Adcy4, Adcy6, Adcy7, Adcy8, Adcy9
mmu04976	Bile secretion	0.000311	71	9	Adcy1, Adcy3, Adcy2, Adcy5, Adcy4, Adcy6, Adcy7, Adcy8, Adcy9
mmu01522	Endocrine resistance	0.000531	95	10	Gper1, Adcy1, Adcy3, Adcy2, Adcy5, Adcy4, Adcy6, Adcy7, Adcy8, Adcy9
mmu04540	Gap junction	2.48E-07	86	14	Lpar1, Gnai1, Adcy1, Adcy3, Adcy2, Adcy5, Drd2, Gnai3, Adcy4, Adcy6, Adcy7, Adcy8, Adcy9, Gnai2
mmu04072	Phospholipase D signaling pathway	6.77E-09	144	20	Lpar2, Grm8, Lpar1, Lpar3, Adcy4, Adcy3, Adcy2, Adcy5, Grm7, Cxcr2, Adcy1, Adcy6, Adcy7, Lpar5, Adcy9, Grm3, Grm2, Adcy8, Cxcr1, Grm6
mmu05150	Staphylococcus aureus infection	0.00093	51	7	Fpr1, Fpr3, Fpr2, C5ar1, C3ar1, C3, Hc
mmu04022	cGMP-PKG signaling pathway	2.51E-06	173	18	Gnai3, Oprd1, Gnai1, Adcy1, Adcy3, Adcy2, Adcy5, Adra2a, Adra2b, Adra2c, Adora1, Adcy4, Adcy6, Adcy7, Adcy8, Adcy9, Bdkrb2, Gnai2
mmu05032	Morphine addiction	1.62E-09	92	17	Oprm1, Gnb1, Gnai1, Adcy1, Adcy3, Adcy2, Adcy5, Gnai3, Arrb2, Adcy4, Adcy6, Adora1, Adcy7, Adcy9, Gng2, Adcy8, Gnai2

mmu04918	Thyroid hormone synthesis	0.000373	73	9	Adcy1, Adcy3, Adcy2, Adcy5, Adcy4, Adcy6, Adcy7, Adcy8, Adcy9
mmu04916	Melanogenesis	7.83E-06	100	13	Gnai3, Pomc, Gnai1, Adcy1, Adcy3, Adcy2, Adcy5, Adcy4, Adcy6, Adcy7, Adcy8, Adcy9, Gnai2
mmu04915	Estrogen signaling pathway	1.10E-06	98	14	Oprm1, Gnai3, Gnai1, Gper1, Adcy1, Adcy3, Adcy2, Adcy5, Adcy4, Adcy6, Adcy7, Adcy8, Adcy9, Gnai2
mmu04914	Progesterone-mediated oocyte maturation	1.28E-05	89	12	Gnai3, Gnai1, Adcy1, Adcy3, Adcy2, Adcy5, Adcy4, Adcy6, Adcy7, Adcy8, Adcy9, Gnai2
mmu04913	Ovarian steroidogenesis	6.93E-05	58	9	Adcy4, Adcy3, Adcy2, Adcy5, Adcy1, Adcy6, Adcy7, Adcy8, Adcy9
mmu04911	Insulin secretion	0.000953	85	9	Adcy1, Adcy3, Adcy2, Adcy5, Adcy4, Adcy6, Adcy7, Adcy8, Adcy9
mmu04713	Circadian entrainment	3.10E-08	98	16	Gnb1, Mtnr1b, Adcy1, Adcy3, Adcy2, Adcy5, Gnai3, Adcy4, Adcy6, Adcy7, Adcy8, Adcy9, Gng2, Mtnr1a, Gnai2, Gnai1

[1] *Count tot* is the number of genes associated to the KEGG pathway; [2]*Net count* is the number of genes associated to the KEGG pathway *and* present in the cluster.

Table S5. Cluster 2 enriched GO terms.

GO_ID	GO_NAME	*p*-Value	Tot Count	Net Count	Genes
GO:0034677	integrin alpha7-beta1 complex	0.000377	2	2	Itgb1, Itga7
GO:0005584	collagen type I trimer	0.000377	2	2	Col1a2, Col1a1
GO:0009986	cell surface	0.000105	213	9	Itgb1, Itgb3, Sdc1, Itga2, Sdc4, Itga5, Itga6, Cd44, Itga1
GO:0007155	cell adhesion	0.000134	45	5	Cd44, Itga1, Itga4, Itgb4, Itga7
GO:0009925	basal plasma membrane	0.000377	11	3	Itga9, Itgb4, Itga6
GO:0031012	extracellular matrix	1.55E-12	39	10	Col5a2, Col5a1, Col1a2, Col4a2, Col4a1, Col1a1, Col6a1, Col3a1, Col6a2, Col12a1
GO:0005178	integrin binding	3.67E-07	28	6	Itgb1, Itgb3, Itgb5, Itgb6, Itga5, Itga6
GO:0005587	collagen type IV trimer	2.43E-12	6	6	Col4a6, Col4a5, Col4a4, Col4a3, Col4a2, Col4a1
GO:0005581	collagen trimer	9.19E-20	11	10	Col10a1, Col11a1, Col11a2, Col5a2, Col5a1, Col1a2, Col1a1, Col3a1, Col6a3, Col2a1
GO:0042383	sarcolemma	0.000217	51	5	Itgb1, Col6a1, Col6a3, Col6a2, Itga7
GO:0005604	basement membrane	2.64E-16	68	14	Col15a1, Col5a1, Itgb4, Col7a1, Col18a1, Itga6, Col4a6, Col4a5, Col4a4, Col4a3, Col28a1, Col4a1, Col2a1, Col4a2
GO:0030056	hemidesmosome	1.86E-05	4	3	Itgb4, Itga6, Col17a1
GO:0030199	collagen fibril organization	2.42E-08	32	7	Col11a1, Col11a2, Col14a1, Col5a2, Col5a1, Col3a1, Col2a1
GO:0030198	extracellular matrix organization	0.000147	80	6	Itga8, Ptk2, Col18a1, Col19a1, Col4a6, Col27a1
GO:0071230	cellular response to amino acid stimulus	9.48E-10	34	8	Col5a2, Col4a6, Col1a2, Col4a1, Col1a1, Col6a1, Col3a1, Col16a1

GO:0006029	proteoglycan metabolic process	0.000377	2	2	Col2a1, Col11a1
GO:0005201	extracellular matrix structural constituent	1.04E-06	7	4	Col4a4, Col27a1, Col5a1, Col1a1
GO:0007160	cell-matrix adhesion	0.000377	30	4	Itgb1, Col5a3, Itga6, Itga2b
GO:0001568	blood vessel development	0.000247	53	5	Col3a1, Ptk2, Col5a1, Itgav, Col1a1
GO:0008305	integrin complex	0.000377	2	2	Itgb4, Itga6
GO:0009897	external side of plasma membrane	2.42E-08	224	13	Itgb1, Itgb3, Itgb6, Itga1, Itga2, Itga3, Itga4, Itga5, Itgav, Cd44, Itga6, Itga2b, Sdc1
GO:0045178	basal part of cell	0.000248	9	3	Itga1, Itga2, Itga6
GO:0005578	proteinaceous extracellular matrix	4.36E-08	114	10	Col10a1, Col11a1, Col1a1, Col5a1, Col26a1, Col6a1, Col3a1, Col6a3, Col6a2, Col2a1

[1] *Count tot* is the number of genes associated to the GO term; [2] *Net count* is the number of genes associated to the GO term *and* present in the cluster.

Table S6. Cluster 2 enriched KEGG pathways.

KEGG_ID	KEGG_Name	*p*-Value	Count_Tot	Count_Net	Genes
mmu05200	Pathways in cancer	3.75E-05	395	13	Itgav, Itgb1, Col4a6, Itga6, Ptk2, Itga2, Col4a5, Itga2b, Col4a2, Col4a1, Col4a4, Col4a3, Itga3
mmu04640	Hematopoietic cell lineage	9.50E-08	86	9	Itga1, Itgb3, Itga6, Itga5, Itga2b, Itga2, Itga4, Cd44, Itga3
mmu05205	Proteoglycans in cancer	2.97E-07	207	12	Col1a2, Col1a1, Itgb1, Itgb3, Itgb5, Itga5, Ptk2, Itga2, Sdc1, Itgav, Cd44, Sdc4
mmu04933	AGE-RAGE signaling pathway in diabetic complications	3.61E-07	102	9	Col1a2, Col1a1, Col4a6, Col4a5, Col3a1, Col4a2, Col4a1, Col4a4, Col4a3
mmu05146	Amoebiasis	6.06E-08	109	10	Col1a2, Col1a1, Col4a6, Ptk2, Col4a5, Col3a1, Col4a2, Col4a1, Col4a4, Col4a3
mmu04512	ECM-receptor interaction	9.55E-63	83	39	Itgb8, Itgb6, Itga7, Itga6, Itga5, Itga2b, Itga2, Itga9, Sdc4, Col1a2, Col1a1, Col9a3, Col9a2, Itga1, Itgb7, Col2a1, Col4a2, Col4a1, Col4a4, Col4a3, Itgav, Itgb1, Itgb3, Itgb5, Sdc1, Itga11, Itga4, Cd44, Itga3, Itga10, Col4a6, Gp6, Itga8, Col6a1, Col4a5, Col6a2, Col6a3, Col9a1, Itgb4
mmu04510	Focal adhesion	6.41E-40	203	36	Itgb8, Itgb6, Itga7, Itga6, Itga5, Itga2b, Itga2, Itga9, Col1a2, Col1a1, Col9a3, Col9a2, Itga1, Ptk2, Itgb7, Col2a1, Col4a2, Col4a1, Col4a4, Col4a3, Itgav, Itgb1, Itgb3, Itgb5, Itga11, Itga4, Itga3, Itga10, Col4a6, Itga8, Col6a1, Col4a5, Col6a2, Col6a3, Col9a1, Itgb4
mmu05222	Small cell lung cancer	4.70E-13	85	13	Itgav, Itgb1, Col4a6, Itga6, Ptk2, Itga2, Col4a5, Itga2b, Col4a2, Col4a1, Col4a4, Col4a3, Itga3
mmu04810	Regulation of actin cytoskeleton	8.07E-17	218	21	Itgav, Itga10, Itgb1, Itgb3, Itga1, Itgb8, Itgb6, Itga8, Itga7, Itga6, Itga5, Itga2b, Itga2, Itgb7, Itga4, Itga9, Ptk2, Itgb5, Itga11, Itga3, Itgb4
mmu04611	Platelet activation	1.66E-05	123	8	Col1a2, Col1a1, Itgb1, Itgb3, Itga2b, Itga2, Col3a1, Gp6

mmu04514	Cell adhesion molecules (CAMs)	2.75E-06	169	10	Itgav, Itgb1, Itgb8, Itga8, Itga6, Itga4, Sdc1, Itgb7, Itga9, Sdc4
mmu04974	Protein digestion and absorption	1.02E-45	90	32	Col1a2, Col1a1, Col9a3, Col9a2, Col24a1, Col17a1, Col18a1, Col3a1, Col2a1, Col4a2, Col4a1, Col4a4, Col4a3, Col22a1, Col14a1, Col15a1, Col27a1, Col4a6, Col10a1, Col11a1, Col11a2, Col12a1, Col13a1, Col5a2, Col6a1, Col4a5, Col5a1, Col7a1, Col5a3, Col6a2, Col6a3, Col9a1
mmu05410	Hypertrophic cardiomyopathy (HCM)	4.44E-24	84	20	Itgav, Itga10, Itgb1, Itgb3, Itga1, Itgb8, Itgb6, Itga8, Itga7, Itga6, Itga5, Itga2b, Itga2, Itgb7, Itga11, Itga9, Itga4, Itgb5, Itga3, Itgb4
mmu05414	Dilated cardiomyopathy	1.06E-23	88	20	Itgav, Itga10, Itgb1, Itgb3, Itga1, Itgb8, Itgb6, Itga8, Itga7, Itga6, Itga5, Itga2b, Itga2, Itgb7, Itga11, Itga9, Itga4, Itgb5, Itga3, Itgb4
mmu05412	Arrhythmogenic right ventricular cardiomyopathy (ARVC)	3.09E-25	74	20	Itgav, Itga10, Itgb1, Itgb3, Itga1, Itgb8, Itgb6, Itga8, Itga7, Itga6, Itga5, Itga2b, Itga2, Itgb7, Itga11, Itga9, Itga4, Itgb5, Itga3, Itgb4
mmu04151	PI3K-Akt signaling pathway	2.02E-31	344	36	Itgb8, Itgb6, Itga7, Itga6, Itga5, Itga2b, Itga2, Itga9, Col1a2, Col1a1, Col9a3, Col9a2, Itga1, Ptk2, Itgb7, Col2a1, Col4a2, Col4a1, Col4a4, Col4a3, Itgav, Itgb1, Itgb3, Itgb5, Itga11, Itga4, Itga3, Itga10, Col4a6, Itga8, Col6a1, Col4a5, Col6a2, Col6a3, Col9a1, Itgb4

[1] *Count tot* is the number of genes associated to the KEGG pathway; [2] *Net count* is the number of genes associated to the KEGG pathway *and* present in the cluster.

Table S7. Cluster 3 enriched GO terms.

GO_ID	GO_Name	*p*-Value	Tot Count	Net Count	Genes
GO:0044212	transcription regulatory region DNA binding	0.000247	87	6	Cebpb, Cebpa, Egr2, Rxra, Klf4, Ncor1
GO:0045444	fat cell differentiation	6.37E-05	67	6	Cebpb, Klf4, Egr2, Cebpa, Med1, Ep300
GO:0003677	DNA binding	3.32E-08	414	16	Hdac1, Cebpb, Cebpa, Ep300, Egr2, Ppargc1a, Rxra, Klf5, Med1, Med6, Ncor1, Ebf1, Ncoa1, Hdac3, Ncor2, Crebbp
GO:0031490	chromatin DNA binding	0.000827	32	4	Hdac3, Ep300, Ppargc1a, Rxra
GO:0050873	brown fat cell differentiation	3.10E-05	31	5	Cebpb, Cebpa, Slc2a4, Fabp4, Adipoq
GO:0045944	positive regulation of transcription from RNA polymerase II promoter	3.10E-05	536	14	Cebpb, Klf4, Ncoa1, Egr2, Wnt10b, Rxra, Cebpa, Ppargc1a, Med10, Med1, Ebf1, Tnf, Ep300, Wnt1
GO:0045892	negative regulation of transcription, DNA-templated	0.000967	235	8	Hdac1, Cebpb, Cebpa, Ncoa2, Fabp4, Adipoq, Ncor2, Ncor1
GO:0045893	positive regulation of transcription, DNA-templated	2.93E-05	296	11	Cebpb, Cebpa, Egr2, Wnt1, Ppargc1a, Klf5, Klf4, Ebf1, Tnf, Ep300, Crebbp
GO:0003682	chromatin binding	1.39E-06	271	12	Ncoa2, Ncoa3, Hdac3, Ncoa1, Cebpb, Egr2, Med12, Med1, Ep300, Ncor2, Crebbp, Hdac1
GO:0051091	positive regulation of sequence-specific DNA binding transcription factor activity	0.000967	35	4	Wnt1, Wnt10b, Ep300, Ppargc1a

GO:0016592	mediator complex	6.32E-55	27	25	Med13l, Med30, Med31, Ccnc, Med21, Med20, Med23, Med24, Med27, Med26, Cdk8, Med1, Med6, Med7, Med4, Med8, Med9, Med10, Med12, Med13, Med14, Med16, Med17, Med18, Med19
GO:0019827	stem cell population maintenance	3.54E-14	59	12	Med21, Med30, Med24, Med27, Med10, Med12, Klf4, Med14, Med7, Med17, Med6, Med15
GO:0008134	transcription factor binding	2.13E-11	101	12	Ncoa2, Hdac1, Med1, Ncoa1, Ep300, Cebpa, Ppargc1a, Rxra, Med12, Klf4, Hdac3, Crebbp
GO:0006355	regulation of transcription, DNA-templated	0.000179	178	8	Ncoa2, Cebpa, Egr2, Rxra, Med1, Ebf1, Ep300, Crebbp
GO:0003713	transcription coactivator activity	3.10E-05	56	6	Ncoa2, Cebpa, Ncoa1, Ppargc1a, Med1, Ep300
GO:0000122	negative regulation of transcription from RNA polymerase II promoter	0.000967	382	10	Hdac1, Ncoa2, Cebpa, Wnt10b, Lep, Klf5, Med1, Tnf, Ncor2, Ncor1

[1] *Count tot* is the number of genes associated to the GO term; [2] *Net count* is the number of genes associated to the GO term *and* present in the cluster.

Table S8. Cluster 3 enriched KEGG pathways.

KEGG_ID	KEGG_Name	*p*-Value	Tot Count	Net Count	Genes
mmu04920	Adipocytokine signaling pathway	0.000503	72	6	Slc2a4, Lep, Adipoq, Rxra, Tnf, Ppargc1a
mmu04919	Thyroid hormone signaling pathway	2.94E-21	117	20	Med12, Ep300, Med16, Med17, Med24, Crebbp, Hdac1, Hdac3, Med4, Med14, Med13, Rxra, Med30, Ncoa1, Ncoa2, Ncoa3, Med13l, Med27, Med1, Ncor1

[1] *Count tot* is the number of genes associated to the KEGG pathway; [2] *Net count* is the number of genes associated to the KEGG pathway *and* present in the cluster.

Table S9. Cluster 4 enriched GO terms.

GO_ID	GO_Name	p-Value	Tot Count	Net Count	Genes
GO:1902379	chemoattractant activity involved in axon guidance	0.000282	2	2	Wnt5a, Wnt7b
GO:0001944	vasculature development	0.000959	23	3	Wnt7b, Fzd5, Lrp5
GO:0021874	Wnt signaling pathway involved in forebrain neuroblast division	0.000282	2	2	Wnt3a, Lrp6
GO:0021871	forebrain regionalization	0.000941	4	2	Wnt2b, Wnt7b
GO:0048103	somatic stem cell division	0.000173	10	3	Wnt3a, Vangl2, Wnt7a
GO:0090263	positive regulation of canonical Wnt signaling pathway	3.52E-06	29	5	Wnt3a, Sfrp2, Wls, Wnt7a, Dvl2
GO:1904938	planar cell polarity pathway involved in axon guidance	5.49E-08	5	4	Wnt5a, Fzd3, Vangl2, Wnt7b
GO:0035426	extracellular matrix-cell signaling	0.000941	4	2	Fzd4, Lrp5
GO:0036514	dopaminergic neuron axon guidance	0.000584	3	2	Vangl2, Fzd3
GO:0016055	Wnt signaling pathway	1.82E-14	38	10	Porcn, Ryk, Lrp5, Fzd8, Wnt2, Wls, Wnt6, Dvl2, Wnt9b, Wnt7b
GO:0009986	cell surface	0.000283	213	7	Wnt3a, Wnt6, Wnt4, Wnt5b, Wnt5a, Wnt7a, Wnt7b
GO:0045893	positive regulation of transcription, DNA-templated	0.000282	296	8	Wnt3a, Fzd4, Lrp5, Wnt6, Wnt4, Lrp6, Wnt5a, Wnt7a

GO:0030514	negative regulation of BMP signaling pathway	0.000149	30	4	Sfrp2, Fzd1, Wnt5a, Cer1
GO:0060071	Wnt signaling pathway, planar cell polarity pathway	1.24E-08	10	5	Vangl2, Sfrp1, Wnt5a, Wnt9b, Fzd6
GO:0072174	metanephric tubule formation	0.000282	2	2	Wnt9b, Wnt4
GO:0060028	convergent extension involved in axis elongation	0.000941	4	2	Wnt5a, Vangl2
GO:0090090	negative regulation of canonical Wnt signaling pathway	8.00E-06	35	5	Sfrp2, Sfrp1, Gpc3, Wnt5a, Wnt11
GO:0061038	uterus morphogenesis	0.000584	3	2	Wnt7a, Wnt9b
GO:0043507	positive regulation of JUN kinase activity	5.76E-06	13	4	Fzd8, Dvl2, Wnt5a, Fzd5
GO:0030901	midbrain development	7.39E-05	25	4	Wnt3a, Wnt5a, Wls, Lrp6
GO:0051091	positive regulation of sequence-specific DNA binding transcription factor activity	0.000238	35	4	Wnt3a, Fzd4, Lrp6, Lrp5
GO:0009948	anterior/posterior axis specification	0.000281	12	3	Wnt3, Wls, Cer1
GO:0022007	convergent extension involved in neural plate elongation	0.000584	3	2	Dvl2, Vangl2

GO:0035567	non-canonical Wnt signaling pathway	1.24E-08	4	4	Wnt5a, Vangl2, Wnt11, Wnt4
GO:0033278	cell proliferation in midbrain	0.000584	3	2	Wnt3a, Wnt2
GO:0035115	embryonic forelimb morphogenesis	0.000941	22	3	Wnt3, Lrp6, Wnt9a
GO:0001934	positive regulation of protein phosphorylation	0.000881	58	4	Wnt3a, Fzd1, Wnt5a, Fzd8
GO:0071481	cellular response to X-ray	0.000584	3	2	Sfrp2, Sfrp1
GO:1902262	apoptotic process involved in patterning of blood vessels	0.000941	4	2	Wnt7b, Lrp5
GO:0005110	frizzled-2 binding	0.000282	2	2	Wnt3a, Wnt5a
GO:0042813	Wnt-activated receptor activity	6.66E-12	8	6	Fzd2, Fzd3, Fzd4, Ryk, Lrp5, Fzd8
GO:0035413	positive regulation of catenin import into nucleus	0.000941	4	2	Wnt3a, Sfrp2
GO:0035019	somatic stem cell population maintenance	0.000173	32	4	Vangl2, Sfrp1, Wnt7a, Lrp5
GO:0019904	protein domain specific binding	0.000732	55	4	Wnt3a, Wnt3, Wnt5a, Dvl2
GO:0005109	frizzled binding	1.68E-12	13	7	Wnt3a, Ryk, Wnt4, Dvl2, Wnt5a, Wnt7a, Wnt7b
GO:0060070	canonical Wnt signaling pathway	2.08E-20	56	14	Wnt3a, Porcn, Lrp6, Fzd5, Lrp5, Fzd8, Wnt2, Wnt4, Dvl2, Fzd1, Fzd4, Wnt5a, Wnt11, Wnt7b
GO:0017147	Wnt-protein binding	1.94E-23	18	12	Sfrp2, Fzd1, Lrp6, Ryk, Fzd6, Lrp5, Fzd8, Wls, Porcn, Fzd4, Fzd10, Fzd5
GO:0060638	mesenchymal-epithelial cell signaling	0.000584	3	2	Wnt2b, Wnt5a

GO:0010628	positive regulation of gene expression	0.000732	177	6	Wnt3, Wnt10a, Wnt6, Wnt8b, Wnt7a, Wnt7b
GO:0036342	post-anal tail morphogenesis	1.24E-08	22	6	Wnt3a, Sfrp2, Fzd3, Lrp6, Wnt5a, Vangl2
GO:2000049	positive regulation of cell-cell adhesion mediated by cadherin	0.000584	3	2	Wnt3a, Wnt5a
GO:0036515	serotonergic neuron axon guidance	0.000584	3	2	Vangl2, Fzd3
GO:0071542	dopaminergic neuron differentiation	0.000512	16	3	Wnt5a, Sfrp1, Lrp6
GO:0009952	anterior/posterior pattern specification	1.67E-05	78	6	Wnt3a, Lrp6, Wnt3, Cer1, Vangl2, Wnt5a
GO:0048697	positive regulation of collateral sprouting in absence of injury	0.000282	2	2	Wnt3, Wnt3a
GO:0005578	proteinaceous extracellular matrix	8.64E-06	114	7	Wnt3, Wnt6, Wnt4, Wnt5b, Wnt5a, Wnt7a, Wnt11
GO:0090179	planar cell polarity pathway involved in neural tube closure	2.74E-06	3	3	Dvl2, Wnt5a, Vangl2
GO:0003401	axis elongation	0.000584	3	2	Wnt5a, Lrp6

[1] *Count tot* is the number of genes associated to the GO term; [2] *Net count* is the number of genes associated to the GO term *and* present in the cluster.

Table S10. Cluster 4 enriched KEGG pathways.

KEGG_ID	KEGG_Name	*p*-Value	Tot Count	Net Count	Genes
mmu05200	Pathways in cancer	7.51E-26	395	27	Dvl2, Wnt10a, Fzd4, Wnt8a, Wnt16, Fzd6, Fzd10, Fzd1, Wnt7b, Fzd3, Wnt6, Wnt7a, Fzd5, Wnt9b, Wnt9a, Wnt2, Fzd2, Wnt11, Wnt4, Wnt3a, Wnt3, Wnt2b, Wnt5b, Wnt5a, Fzd9, Fzd8, Wnt8b
mmu05205	Proteoglycans in cancer	1.46E-33	207	27	Gpc3, Wnt10a, Fzd4, Wnt8a, Wnt16, Fzd6, Fzd10, Fzd1, Wnt7b, Fzd3, Wnt6, Wnt7a, Fzd5, Wnt9b, Wnt9a, Wnt2, Fzd2, Wnt11, Wnt4, Wnt3a, Wnt3, Wnt2b, Wnt5b, Wnt5a, Fzd9, Fzd8, Wnt8b
mmu05166	HTLV-I infection	1.05E-29	285	27	Dvl2, Wnt10a, Fzd4, Wnt8a, Wnt16, Fzd6, Fzd10, Fzd1, Wnt7b, Fzd3, Wnt6, Wnt7a, Fzd5, Wnt9b, Wnt9a, Wnt2, Fzd2, Wnt11, Wnt4, Wnt3a, Wnt3, Wnt2b, Wnt5b, Wnt5a, Fzd9, Fzd8, Wnt8b
mmu05224	Breast cancer	6.15E-42	146	29	Dvl2, Wnt10a, Fzd4, Wnt8a, Wnt16, Fzd6, Fzd10, Fzd1, Wnt7b, Fzd3, Wnt6, Wnt7a, Fzd5, Wnt9b, Wnt9a, Wnt2, Fzd2, Wnt11, Wnt4, Wnt3a, Wnt3, Wnt2b, Lrp5, Wnt5b, Wnt5a, Lrp6, Fzd9, Fzd8, Wnt8b
mmu04550	Signaling pathways regulating pluripotency of stem cells	2.29E-38	140	27	Dvl2, Wnt10a, Fzd4, Wnt8a, Wnt16, Fzd6, Fzd10, Fzd1, Wnt7b, Fzd3, Wnt6, Wnt7a, Fzd5, Wnt9b, Wnt9a, Wnt2, Fzd2, Wnt11, Wnt4, Wnt3a, Wnt3, Wnt2b, Wnt5b, Wnt5a, Fzd9, Fzd8, Wnt8b
mmu04390	Hippo signaling pathway	3.25E-37	154	27	Dvl2, Wnt10a, Fzd4, Wnt8a, Wnt16, Fzd6, Fzd10, Fzd1, Wnt7b, Fzd3, Wnt6, Wnt7a, Fzd5, Wnt9b, Wnt9a, Wnt2, Fzd2, Wnt11, Wnt4, Wnt3a, Wnt3, Wnt2b, Wnt5b, Wnt5a, Fzd9, Fzd8, Wnt8b
mmu04310	Wnt signaling pathway	6.36E-61	146	37	Wnt5a, Dvl2, Fzd3, Fzd4, Wnt8a, Wnt16, Fzd6, Wnt10a, Sfrp1, Fzd10, Fzd1, Wnt7b, Sfrp4, Wnt6, Wnt7a, Wif1, Fzd5, Sfrp2, Vangl2, Wnt9b, Sfrp5, Wnt3a, Wnt2, Fzd2, Wnt11, Wnt4, Wnt9a, Wnt3, Wnt2b, Lrp5, Wnt5b, Porcn, Cer1, Lrp6, Fzd9, Fzd8, Wnt8b

mmu05217	Basal cell carcinoma	5.48E-51	55	27	Dvl2, Wnt10a, Fzd4, Wnt8a, Wnt16, Fzd6, Fzd10, Fzd1, Wnt7b, Fzd3, Wnt6, Wnt7a, Fzd5, Wnt9b, Wnt9a, Wnt2, Fzd2, Wnt11, Wnt4, Wnt3a, Wnt3, Wnt2b, Wnt5b, Wnt5a, Fzd9, Fzd8, Wnt8b
mmu04916	Melanogenesis	1.70E-42	100	27	Dvl2, Wnt10a, Fzd4, Wnt8a, Wnt16, Fzd6, Fzd10, Fzd1, Wnt7b, Fzd3, Wnt6, Wnt7a, Fzd5, Wnt9b, Wnt9a, Wnt2, Fzd2, Wnt11, Wnt4, Wnt3a, Wnt3, Wnt2b, Wnt5b, Wnt5a, Fzd9, Fzd8, Wnt8b
mmu04150	mTOR signaling pathway	4.06E-41	156	29	Dvl2, Wnt10a, Fzd4, Wnt8a, Wnt16, Fzd6, Fzd10, Fzd1, Wnt7b, Fzd3, Wnt6, Wnt7a, Fzd5, Wnt9b, Wnt9a, Wnt2, Fzd2, Wnt11, Wnt4, Wnt3a, Wnt3, Wnt2b, Lrp5, Wnt5b, Wnt5a, Lrp6, Fzd9, Fzd8, Wnt8b

[1] *Count tot* is the number of genes associated to the KEGG pathway; [2] *Net count* is the number of genes associated to the KEGG pathway *and* present in the cluster.

Table S11. Cluster 5 enriched GO terms.

GO_ID	GO_Name	*p*-Value	Tot Count	Net Count	Genes
GO:0018108	peptidyl-tyrosine phosphorylation	3.23E-06	38	5	Pdgfrb, Pdgfra, Tek, Csf1r, Kit
GO:0048557	embryonic digestive tract morphogenesis	0.000165	15	3	Pdgfra, Fgf10, Fgfr2
GO:0060129	thyroid-stimulating hormone-secreting cell differentiation	0.000594	4	2	Fgf2, Fgf8
GO:0060128	corticotropin hormone secreting cell differentiation	0.00032	3	2	Fgf2, Fgf8
GO:0048286	lung alveolus development	9.15E-05	38	4	Flt4, Fgf10, Fgfr2, Kdr
GO:0001759	organ induction	3.17E-07	8	4	Fgfr4, Fgf2, Fgf1, Fgfr1
GO:0048514	blood vessel morphogenesis	8.50E-07	29	5	Flt1, Flt4, Fgf10, Epha2, Fgfr1
GO:0008544	epidermis development	4.52E-05	31	4	Igf1r, Fgf10, Egfr, Insr
GO:0008543	fibroblast growth factor receptor signaling pathway	1.15E-07	18	5	Fgfr2, Fgf17, Fgfr3, Fgf9, Fgf8
GO:0090263	positive regulation of canonical Wnt signaling pathway	8.50E-07	29	5	Fgf10, Fgfr2, Fgfr3, Fgf9, Fgf2
GO:0030238	male sex determination	3.66E-05	8	3	Igf1r, Insr, Fgf9
GO:0007267	cell-cell signaling	0.000319	19	3	Fgf10, Fgfr2, Fgf9
GO:0046777	protein autophosphorylation	1.85E-05	55	5	Met, Pdgfra, Csf1r, Egfr, Pdgfrb
GO:0009986	cell surface	5.32E-05	213	7	Pdgfrb, Pdgfra, Egfr, Csf1r, Kit, Ngfr, Kdr
GO:0017134	fibroblast growth factor binding	2.58E-05	7	3	Gpc1, Fgfr2, Fgfr1
GO:0030324	lung development	4.87E-14	75	11	Pdgfra, Fgf18, Kdr, Fgf9, Fgf8, Fgfr4, Fgf10, Fgfr2, Fgf2, Fgf1, Fgfr1
GO:0060665	regulation of branching involved in salivary gland morphogenesis by	3.18E-08	5	4	Fgf7, Met, Fgf10, Fgfr1

	mesenchymal-epithelial signaling				
GO:0060667	branch elongation involved in salivary gland morphogenesis	0.000143	2	2	Fgf10, Fgfr2
GO:0002053	positive regulation of mesenchymal cell proliferation	0.000594	25	3	Kdr, Fgf9, Fgfr1
GO:0005021	vascular endothelial growth factor-activated receptor activity	0.00032	3	2	Kdr, Flt1
GO:0009887	animal organ morphogenesis	3.70E-05	67	5	Pdgfra, Igf1r, Fgf10, Fgfr2, Insr
GO:0023019	signal transduction involved in regulation of gene expression	0.000165	15	3	Pdgfra, Fgf5, Fgf8
GO:0030949	positive regulation of vascular endothelial growth factor receptor signaling pathway	9.10E-06	5	3	Fgf18, Fgf10, Fgf9
GO:0042472	inner ear morphogenesis	0.000248	53	4	Fgf10, Fgfr2, Fgf9, Fgfr1
GO:0048146	positive regulation of fibroblast proliferation	0.000655	26	3	Fgf10, Egfr, Ngfr
GO:0001934	positive regulation of protein phosphorylation	0.00032	58	4	Fgf4, Fgf2, Fgf1, Tek
GO:0048565	digestive tract development	0.00043	22	3	Fgf10, Fgfr2, Kit
GO:0050679	positive regulation of epithelial cell proliferation	5.32E-10	32	7	Egfr, Kdr, Fgf9, Fgf7, Fgf10, Fgf2, Fgf1
GO:0005007	fibroblast growth factor-activated receptor activity	0.00032	3	2	Fgfr3, Fgfr1

GO:0035607	fibroblast growth factor receptor signaling pathway involved in orbitofrontal cortex development	0.000143	2	2	Fgfr2, Fgfr1
GO:0060595	fibroblast growth factor receptor signaling pathway involved in mammary gland specification	0.000143	2	2	Fgf10, Fgfr2
GO:0021847	ventricular zone neuroblast division	0.00032	3	2	Fgfr2, Fgfr1
GO:0038083	peptidyl-tyrosine autophosphorylation	2.58E-05	7	3	Igf1r, Insr, Kdr
GO:0031069	hair follicle morphogenesis	4.52E-05	31	4	Fgf7, Fgf10, Fgfr2, Ngfr
GO:0008284	positive regulation of cell proliferation	4.71E-12	165	12	Fgf10, Fgf17, Csf1r, Egfr, Fgf18, Fgf9, Fgf8, Fgf7, Fgf4, Fgfr2, Fgf2, Fgfr1
GO:0045840	positive regulation of mitotic nuclear division	7.35E-07	10	4	Pdgfrb, Igf1r, Insr, Fgf8
GO:0060615	mammary gland bud formation	0.000143	2	2	Fgf10, Fgfr2
GO:0060445	branching involved in salivary gland morphogenesis	2.62E-08	13	5	Fgf7, Fgf10, Fgfr2, Fgfr1, Fgf8
GO:0000165	MAPK cascade	0.000896	29	3	Fgf23, Fgfr3, Fgf8
GO:0010628	positive regulation of gene expression	0.000173	177	6	Kit, Fgf9, Fgf8, Fgf4, Fgfr2, Fgf2
GO:0070374	positive regulation of ERK1 and ERK2 cascade	4.79E-07	52	6	Pdgfrb, Csf1r, Fgf2, Fgf18, Fgf10, Fgf23
GO:0001525	angiogenesis	9.15E-05	84	5	Fgf2, Tek, Fgf18, Fgf9, Flt1
GO:0030097	hemopoiesis	0.000453	65	4	Pdgfrb, Tek, Kdr, Kit
GO:0021762	substantia nigra development	0.000143	2	2	Fgf2, Fgfr3
GO:0021769	orbitofrontal cortex development	0.000143	2	2	Fgfr2, Fgfr1

GO:0007435	salivary gland morphogenesis	3.66E-05	8	3	Fgf10, Egfr, Fgfr1

[1] *Count tot* is the number of genes associated to the GO term; [2] *Net count* is the number of genes associated to the GO term *and* present in the cluster.

Table S12. Cluster 5 enriched KEGG pathways.

KEGG_ID	KEGG_Name	*p*-Value	Count Tot	Net Count	Genes
mmu05200	Pathways in cancer	9.85E-23	395	23	Fgf8, Fgf7, Fgf17, Fgf2, Fgf18, Fgf4, Fgf6, Fgf5, Fgfr3, Fgf9, Fgfr1, Fgfr2, Kit, Fgf23, Fgf20, Igf1r, Fgf1, Fgf10, Pdgfrb, Pdgfra, Met, Egfr, Csf1r
mmu05205	Proteoglycans in cancer	5.88E-05	207	7	Fgfr1, Kdr, Fgf2, Gpc1, Met, Egfr, Igf1r
mmu05224	Breast cancer	8.18E-23	146	18	Fgf8, Fgf7, Fgf23, Fgf9, Fgfr1, Egfr, Fgf17, Fgf2, Fgf18, Fgf4, Kit, Fgf6, Fgf5, Flt4, Fgf1, Fgf20, Fgf10, Igf1r
mmu04510	Focal adhesion	4.99E-06	203	8	Flt1, Flt4, Kdr, Igf1r, Met, Pdgfrb, Pdgfra, Egfr
mmu04015	Rap1 signaling pathway	5.72E-47	215	31	Fgf8, Fgf7, Fgf17, Fgf2, Fgf18, Fgf4, Fgf6, Fgf5, Insr, Fgfr3, Fgfr4, Fgf9, Fgfr1, Fgfr2, Kit, Kdr, Fgf23, Fgf20, Igf1r, Fgf1, Fgf10, Pdgfrb, Pdgfra, Flt1, Flt4, Met, Ngfr, Epha2, Tek, Egfr, Csf1r
mmu04060	Cytokine-cytokine receptor interaction	2.58E-07	264	10	Flt1, Flt4, Kdr, Kit, Ngfr, Met, Pdgfrb, Pdgfra, Egfr, Csf1r
mmu05230	Central carbon metabolism in cancer	8.70E-10	66	8	Fgfr3, Fgfr1, Fgfr2, Met, Kit, Pdgfrb, Pdgfra, Egfr
mmu04520	Adherens junction	4.46E-05	74	5	Met, Igf1r, Egfr, Fgfr1, Insr
mmu04810	Regulation of actin cytoskeleton	5.46E-23	218	20	Fgf8, Fgf7, Fgfr4, Fgf23, Fgf9, Fgfr1, Fgfr2, Fgf17, Fgf2, Fgf18, Fgf4, Fgf6, Fgf5, Egfr, Fgfr3, Fgf1, Pdgfra, Fgf20, Fgf10, Pdgfrb
mmu04010	MAPK signaling pathway	6.67E-22	252	20	Fgf8, Fgf7, Fgfr4, Fgf23, Fgf9, Fgfr1, Fgfr2, Fgf17, Fgf2, Fgf18, Fgf4, Fgf6, Fgf5, Egfr, Fgfr3, Fgf1, Pdgfra, Fgf20, Fgf10, Pdgfrb
mmu01521	EGFR tyrosine kinase inhibitor resistance	1.44E-10	82	9	Fgfr3, Fgfr2, Kdr, Fgf2, Met, Pdgfrb, Pdgfra, Egfr, Igf1r
mmu04550	Signaling pathways regulating pluripotency of stem cells	6.49E-05	140	6	Fgfr3, Fgfr4, Fgfr1, Fgfr2, Fgf2, Igf1r

mmu04066	HIF-1 signaling pathway	0.000244	109	5	Flt1, Tek, Igf1r, Egfr, Insr
mmu05218	Melanoma	4.51E-31	71	19	Fgf8, Fgf7, Fgf23, Fgf9, Fgfr1, Egfr, Fgf17, Fgf2, Fgf18, Fgf4, Fgf6, Fgf5, Met, Fgf1, Pdgfra, Fgf20, Fgf10, Igf1r, Pdgfrb
mmu04072	Phospholipase D signaling pathway	0.000818	144	5	Pdgfra, Egfr, Kit, Insr, Pdgfrb
mmu04014	Ras signaling pathway	2.70E-46	230	31	Fgf8, Fgf7, Fgf17, Fgf2, Fgf18, Fgf4, Fgf6, Fgf5, Insr, Fgfr3, Fgfr4, Fgf9, Fgfr1, Fgfr2, Kit, Kdr, Fgf23, Fgf20, Igf1r, Fgf1, Fgf10, Pdgfrb, Pdgfra, Flt1, Flt4, Met, Ngfr, Epha2, Tek, Egfr, Csf1r
mmu04144	Endocytosis	4.29E-08	286	11	Fgfr3, Flt1, Fgfr4, Fgfr2, Kdr, Igf1r, Kit, Met, Pdgfra, Egfr, Csf1r
mmu05215	Prostate cancer	5.64E-06	89	6	Fgfr1, Fgfr2, Igf1r, Pdgfrb, Pdgfra, Egfr
mmu05214	Glioma	0.000456	66	4	Igf1r, Egfr, Pdgfrb, Pdgfra
mmu04151	PI3K-Akt signaling pathway	9.61E-41	344	31	Fgf8, Fgf7, Fgf17, Fgf2, Fgf18, Fgf4, Fgf6, Fgf5, Insr, Fgfr3, Fgfr4, Fgf9, Fgfr1, Fgfr2, Kit, Kdr, Fgf23, Fgf20, Igf1r, Fgf1, Fgf10, Pdgfrb, Pdgfra, Flt1, Flt4, Met, Ngfr, Epha2, Tek, Egfr, Csf1r

[1] *Count tot* is the number of genes associated to the KEGG pathway; [2] *Net count* is the number of genes associated to the KEGG pathway *and* present in the cluster.

Table S13. Cluster 6 enriched GO terms.

GO_ID	GO_Name	*p*-Value	Count Tot	Net Count	Genes
GO:0071803	positive regulation of podosome assembly	0.000421	5	2	Mapk8, Mapk9
GO:0045944	positive regulation of transcription from RNA polymerase II promoter	5.21E-05	536	7	Sirt1, Fos, Fosb, Junb, Jun, Jund, Mapk14
GO:0004705	JUN kinase activity	5.55E-05	2	2	Mapk8, Mapk10
GO:0035994	response to muscle stretch	9.46E-06	9	3	Mapk14, Jun, Fos
GO:0071277	cellular response to calcium ion	1.48E-09	18	5	Fosb, Junb, Jun, Jund, Fos
GO:0000978	RNA polymerase II core promoter proximal region sequence-specific DNA binding	0.000443	142	4	Fosb, Junb, Jund, Fos
GO:0006468	protein phosphorylation	0.000507	152	4	Mapk14, Mapk8, Mapk9, Mapk10
GO:0042752	regulation of circadian rhythm	0.000421	42	3	Mapk8, Mapk9, Mapk10
GO:0006355	regulation of transcription, DNA-templated	0.000837	178	4	Mapk14, Junb, Jun, Fos

[1] *Count tot* is the number of genes associated to the GO term; [2] *Net count* is the number of genes associated to the GO term *and* present in the cluster.

Table S14. Cluster 6 enriched KEGG pathways.

KEGG_ID	KEGG_Name	*p*-Value	Count Tot	Net Count	Genes
mmu04024	cAMP signaling pathway	2.08E-05	199	5	Jun, Mapk9, Mapk10, Mapk8, Fos
mmu05200	Pathways in cancer	0.000402	395	5	Jun, Mapk9, Mapk10, Mapk8, Fos
mmu04668	TNF signaling pathway	1.61E-15	109	10	Jun, Junb, Mapk13, Mapk10, Mapk11, Mapk14, Mapk9, Fos, Mapk8, Mapk12
mmu04920	Adipocytokine signaling pathway	0.000299	72	3	Mapk9, Mapk10, Mapk8
mmu04370	VEGF signaling pathway	4.54E-06	60	4	Mapk14, Mapk13, Mapk11, Mapk12
mmu04932	Non-alcoholic fatty liver disease (NAFLD)	0.000135	153	4	Jun, Mapk9, Mapk10, Mapk8
mmu05212	Pancreatic cancer	0.000236	66	3	Mapk9, Mapk10, Mapk8
mmu05205	Proteoglycans in cancer	0.000399	207	4	Mapk14, Mapk13, Mapk11, Mapk12
mmu04931	Insulin resistance	0.00097	111	3	Mapk9, Mapk10, Mapk8
mmu04933	AGE-RAGE signaling pathway in diabetic complications	4.66E-12	102	8	Jun, Mapk14, Mapk13, Mapk10, Mapk11, Mapk9, Mapk8, Mapk12
mmu05164	Influenza A	1.73E-10	170	8	Jun, Mapk14, Mapk13, Mapk10, Mapk11, Mapk9, Mapk8, Mapk12
mmu04670	Leukocyte transendothelial migration	6.17E-05	121	4	Mapk14, Mapk13, Mapk11, Mapk12
mmu05161	Hepatitis B	4.77E-06	145	5	Jun, Mapk9, Mapk10, Mapk8, Fos
mmu04380	Osteoclast differentiation	2.57E-19	132	12	Jun, Junb, Mapk13, Mapk10, Mapk11, Mapk14, Mapk9, Fos,

					Fosb, Jund, Mapk12, Mapk8
mmu05169	Epstein-Barr virus infection	1.07E-09	220	8	Jun, Mapk14, Mapk13, Mapk10, Mapk11, Mapk9, Mapk8, Mapk12
mmu05168	Herpes simplex infection	2.95E-05	215	5	Jun, Mapk9, Mapk10, Mapk8, Fos
mmu04071	Sphingolipid signaling pathway	9.86E-10	124	7	Mapk14, Mapk13, Mapk10, Mapk11, Mapk9, Mapk8, Mapk12
mmu04750	Inflammatory mediator regulation of TRP channels	1.07E-09	127	7	Mapk14, Mapk13, Mapk10, Mapk11, Mapk9, Mapk8, Mapk12
mmu04510	Focal adhesion	0.000378	203	4	Jun, Mapk9, Mapk10, Mapk8
mmu04015	Rap1 signaling pathway	0.000444	215	4	Mapk14, Mapk13, Mapk11, Mapk12
mmu04660	T cell receptor signaling pathway	3.31E-10	105	7	Jun, Mapk14, Mapk13, Mapk11, Mapk9, Fos, Mapk12
mmu04723	Retrograde endocannabinoid signaling	2.82E-10	102	7	Mapk14, Mapk13, Mapk10, Mapk11, Mapk9, Mapk8, Mapk12
mmu04215	Apoptosis - multiple species	3.18E-05	32	3	Mapk9, Mapk10, Mapk8
mmu04210	Apoptosis	3.97E-06	138	5	Jun, Mapk9, Mapk10, Mapk8, Fos
mmu04068	FoxO signaling pathway	2.81E-11	134	8	Mapk14, Mapk13, Mapk10, Mapk11, Mapk9, Mapk8, Sirt1, Mapk12
mmu04722	Neurotrophin signaling pathway	1.83E-11	123	8	Jun, Mapk14, Mapk13, Mapk10, Mapk11, Mapk9, Mapk8, Mapk12
mmu04728	Dopaminergic synapse	2.81E-11	134	8	Mapk14, Mapk13, Mapk10, Mapk11,

					Mapk9, Fos, Mapk8, Mapk12
mmu04611	Platelet activation	6.43E-05	123	4	Mapk14, Mapk13, Mapk11, Mapk12
mmu04930	Type II diabetes mellitus	0.000112	50	3	Mapk9, Mapk10, Mapk8
mmu05132	Salmonella infection	6.85E-15	78	9	Jun, Mapk14, Mapk13, Mapk10, Mapk11, Mapk9, Fos, Mapk8, Mapk12
mmu05231	Choline metabolism in cancer	8.93E-07	101	5	Jun, Mapk9, Mapk10, Mapk8, Fos
mmu05133	Pertussis	6.30E-15	75	9	Jun, Mapk14, Mapk13, Mapk10, Mapk11, Mapk9, Fos, Mapk8, Mapk12
mmu04012	ErbB signaling pathway	1.85E-05	87	4	Jun, Mapk9, Mapk10, Mapk8
mmu04010	MAPK signaling pathway	1.71E-12	252	10	Jun, Mapk14, Mapk13, Mapk10, Mapk11, Mapk9, Fos, Jund, Mapk12, Mapk8
mmu04261	Adrenergic signaling in cardiomyocytes	0.000134	152	4	Mapk14, Mapk13, Mapk11, Mapk12
mmu01522	Endocrine resistance	3.51E-14	95	9	Jun, Mapk14, Mapk13, Mapk10, Mapk11, Mapk9, Fos, Mapk8, Mapk12
mmu05031	Amphetamine addiction	7.10E-06	68	4	Jun, Fosb, Fos, Sirt1
mmu04550	Signaling pathways regulating pluripotency of stem cells	0.000104	140	4	Mapk14, Mapk13, Mapk11, Mapk12
mmu05160	Hepatitis C	1.62E-09	136	7	Mapk14, Mapk13, Mapk10, Mapk11, Mapk9, Mapk8, Mapk12

mmu04621	NOD-like receptor signaling pathway	7.89E-12	58	7	Mapk14, Mapk13, Mapk10, Mapk11, Mapk9, Mapk8, Mapk12
mmu04622	RIG-I-like receptor signaling pathway	2.17E-11	68	7	Mapk14, Mapk13, Mapk10, Mapk11, Mapk9, Mapk8, Mapk12
mmu05152	Tuberculosis	9.95E-09	177	7	Mapk14, Mapk13, Mapk10, Mapk11, Mapk9, Mapk8, Mapk12
mmu04310	Wnt signaling pathway	0.000117	146	4	Jun, Mapk9, Mapk10, Mapk8
mmu05014	Amyotrophic lateral sclerosis (ALS)	2.70E-06	52	4	Mapk14, Mapk13, Mapk11, Mapk12
mmu05210	Colorectal cancer	9.17E-08	64	5	Jun, Mapk9, Mapk10, Mapk8, Fos
mmu04664	Fc epsilon RI signaling pathway	2.25E-11	69	7	Mapk14, Mapk13, Mapk10, Mapk11, Mapk9, Mapk8, Mapk12
mmu04917	Prolactin signaling pathway	4.47E-13	74	8	Mapk14, Mapk13, Mapk10, Mapk11, Mapk9, Fos, Mapk8, Mapk12
mmu04914	Progesterone-mediated oocyte maturation	1.18E-10	89	7	Mapk14, Mapk13, Mapk10, Mapk11, Mapk9, Mapk8, Mapk12
mmu04912	GnRH signaling pathway	1.71E-12	89	8	Jun, Mapk14, Mapk13, Mapk10, Mapk11, Mapk9, Mapk8, Mapk12
mmu05145	Toxoplasmosis	5.33E-10	113	7	Mapk14, Mapk13, Mapk10, Mapk11, Mapk9, Mapk8, Mapk12

mmu05142	Chagas disease (American trypanosomiasis)	5.33E-14	103	9	Jun, Mapk14, Mapk13, Mapk10, Mapk11, Mapk9, Fos, Mapk8, Mapk12
mmu05140	Leishmaniasis	1.12E-09	65	6	Jun, Mapk14, Mapk13, Mapk11, Fos, Mapk12
mmu04620	Toll-like receptor signaling pathway	5.18E-14	101	9	Jun, Mapk14, Mapk13, Mapk10, Mapk11, Mapk9, Fos, Mapk8, Mapk12

[1] *Count tot* is the number of genes associated to the KEGG pathway; [2] *Net count* is the number of genes associated to the KEGG pathway *and* present in the cluster.

Table S15. Cluster 8 enriched GO terms

GO_ID	GO_Name	*p*-Value	Count Tot	Net Count	Genes
GO:0004619	phosphoglycerate mutase activity	1.54E-06	2	2	Pgam2, Pgam1
GO:0006096	glycolytic process	6.39E-08	8	3	Pgam2, Gapdhs, Pgk1

[1] *Count tot* is the number of genes associated to the GO term; [2] *Net count* is the number of genes associated to the GO term *and* present in the cluster.

Table S16. Cluster 8 enriched KEGG pathways.

KEGG_ID	KEGG_Name	*p*-Value	Count Tot	Net Count	Genes
mmu00260	Glycine, serine and threonine metabolism	4.70E-06	40	3	Pgam2, Pgam1, Bpgm
mmu00010	Glycolysis / Gluconeogenesis	2.72E-10	66	5	Pgk1, Gapdhs, Pgam1, Pgam2, Bpgm
mmu01100	Metabolic pathways	0.000181	1292	5	Pgk1, Gapdhs, Pgam1, Pgam2, Bpgm
mmu01200	Carbon metabolism	6.25E-05	118	3	Pgk1, Pgam2, Pgam1
mmu05230	Central carbon metabolism in cancer	0.000892	66	2	Pgam2, Pgam1
mmu01230	Biosynthesis of amino acids	2.39E-05	78	3	Pgk1, Pgam2, Pgam1

[1] *Count tot* is the number of genes associated to the KEGG pathway; [2] *Net count* is the number of genes associated to the KEGG pathway *and* present in the cluster.

Table S17. KEGG pathways related to the wound healing and to at least one bridge node.

KEGG ID	Name
mmu04024	cAMP signaling pathway
mmu04370	VEGF signaling pathway
mmu04670	Leukocyte transendothelial migration
mmu04750	Inflammatory mediator regulation of TRP channels
mmu04660	T cell receptor signaling pathway
mmu04662	B cell receptor signaling pathway
mmu04668	TNF signaling pathway
mmu04062	Chemokine signaling pathway
mmu04060	Cytokine-cytokine receptor interaction
mmu04210	Apoptosis
mmu04064	NF-kappa B signaling pathway
mmu04330	Notch signaling pathway
mmu04630	Jak-STAT signaling pathway
mmu04020	Calcium signaling pathway
mmu04310	Wnt signaling pathway

Table S18. GO terms related to the wound healing and to at least one bridge node.

GO term	Name
GO:0019221	cytokine-mediated signaling pathway
GO:0008009	chemokine activity
GO:0030225	macrophage differentiation
GO:0030224	monocyte differentiation
GO:0031012	extracellular matrix
GO:0048103	somatic stem cell division
GO:0042307	positive regulation of protein import into nucleus
GO:0048008	platelet-derived growth factor receptor signaling pathway
GO:0023019	signal transduction involved in regulation of gene expression
GO:0010628	positive regulation of gene expression
GO:0050921	positive regulation of chemotaxis
GO:0004871	signal transducer activity
GO:0060326	cell chemotaxis
GO:0002532	production of molecular mediator involved in inflammatory response
GO:0008544	epidermis development
GO:0016493	C-C chemokine receptor activity
GO:0050728	negative regulation of inflammatory response
GO:0050729	positive regulation of inflammatory response
GO:0001707	mesoderm formation
GO:0071356	cellular response to tumor necrosis factor
GO:0060638	mesenchymal-epithelial cell signaling
GO:0050727	regulation of inflammatory response
GO:2000111	positive regulation of macrophage apoptotic process
GO:2000319	regulation of T-helper 17 cell differentiation
GO:0005031	tumor necrosis factor-activated receptor activity
GO:0030183	B cell differentiation
GO:0010468	regulation of gene expression
GO:0032720	negative regulation of tumor necrosis factor production
GO:0043123	positive regulation of I-kappaB kinase/NF-kappaB signaling
GO:0030595	leukocyte chemotaxis
GO:0006935	chemotaxis
GO:0004950	chemokine receptor activity
GO:0019827	stem cell population maintenance
GO:2000481	positive regulation of cAMP-dependent protein kinase activity
GO:0010934	macrophage cytokine production
GO:0042060	wound healing
GO:0006952	defense response
GO:0006954	inflammatory response
GO:0007267	cell-cell signaling
GO:0072678	T cell migration
GO:0051092	positive regulation of NF-kappaB transcription factor activity
GO:0003700	transcription factor activity, sequence-specific DNA binding
GO:0030198	extracellular matrix organization
GO:0043588	skin development
GO:0005667	transcription factor complex

GO:0005006	epidermal growth factor-activated receptor activity
GO:0007165	signal transduction

Table S19. Bridge node score S.

BRIDGE	Score
Nfkb1	1
Rela	1
Tnfrsf1a	1
Pdgfra	0.993
Jun	0.914
Pdgfrb	0.857
Egfr	0.857
Mapk9	0.857
Mapk14	0.828
Sirt1	0.8
Mapk8	0.771
Tnfrsf1b	0.714
Mapk10	0.714
Mapk11	0.714
Mapk12	0.714
Mapk13	0.714
Ptk2	0.543
Hdac1	0.486
Adam17	0.486
Ngfr	0.486
Arrb2	0.486
Tradd	0.429
Vangl2	0.314
Klf5	0.2
Insr	0.2
Igf1r	0.2
Wls	0.2
Slc2a4	0.2
Met	0.143
Flt1	0.143
Sdc4	0
Gpc1	0
Gpc3	0
Ap2m1	0
Ap2s1	0
Sdc1	0
Ap2b1	0
Psme2	0
Ap2a1	0
Ap2a2	0
Gm3839	0
Fosb	0
Psme3	0
Psme1	0
Cltc	0

Table S20. Ordered list of genes changed over the simulations.

Gene	
1.	Bag4
2.	Pik3r1
3.	Pik3cb
4.	Map2k6
5.	Map3k7
6.	Mapk10
7.	Mapk11
8.	Pik3ca
9.	Map3k14
10.	Atf2
11.	Fadd
12.	Creb3l4
13.	Akt3
14.	Ikbkb
15.	Pik3r5
16.	Map2k4
17.	Rps6ka5
18.	Casp7
19.	Cebpb
20.	Creb3l1
21.	Mapk3
22.	Dab2ip
23.	Map2k1
24.	Jun
25.	Mapk1
26.	Pik3cd
27.	Pik3r3
28.	Dnm1l
29.	Pik3r2
30.	Akt1
31.	Atf6b
32.	Mapk13
33.	Mapk9
34.	Creb3
35.	Creb5
36.	Map2k3
37.	Map2k7
38.	Pik3cg
39.	Atf4
40.	Mapk12
41.	Chuk
42.	Creb1
43.	Pgam5
44.	Rps6ka4
45.	Tnfrsf1a

46.	Tnfrsf1b
47.	Ripk3
48.	Akt2
49.	Creb3l2
50.	Traf2
51.	Casp3
52.	Mlkl
53.	Mapk14
54.	Itch
55.	Nfkbia
56.	Ripk1
57.	Casp8
58.	Cflar
59.	Mapk8
60.	Birc3
61.	Tradd
62.	Creb3l3
63.	Birc2
64.	Ikbkg
65.	Lta
66.	Map3k5
67.	Map3k8
68.	Traf5

Table S21. Table to convert KEGG associations into Boolean rules.

KEGG Biological Relationship	Symbol	Boolean Rule
Activation	-->	AND
Inhibition	--|	NOT
Expression	-->	AND
Repression	--|	NOT
Indirect effect	..>	AND
State change	…	AND
Binding/association	--	AND
Dissociation	-+-	NOT
Phosphorylation	+p	AND
Dephosphorylation	-p	NOT
Glycosylation	+g	AND
Ubiquitination	+u	AND
Methylation	+m	AND

Method S1. Input gene panel setup.

To obtain the panel of input genes, we first performed two queries in PubMed in December 2016, and then we read the retrieved papers in order to find candidate genes to design our panel. The first query, "*wound healing[Title/Abstract]) AND gene expression[Title/Abstract]) AND murine[Title/Abstract]*" resulted in three interesting publication out of 77 [1–3]. Based on the results we selected *Tnf*, *Cxcl2*(MIP-2) and *Wnt* genes. The second query *(wound healing[Title/Abstract]) AND gene expression[Title/Abstract]) AND MOUSE[Title/Abstract])* resulted in two interesting publications out of 187 [3,4]. This allowed to add to the input gene *Fosb*. The selection of such genes out the genes mentioned in the retrieved papers was also based on further analysis of the genes found in the publications by using the repositories GenBank [5], Uniprot [6] and KEGG [7]. This allowed us to retain an important study on wound healing [8], in which are reported the genes whose expression is influenced by the wound heling process. From this study, we selected *Cxcl2*, *Tnf* and *Fgf5*. Finally, we focused our gene search on the genes present on Mouse Wound Healing RT^2 Profiler™ PCR Array (SABiosciences, QIAGEN [9]). This array profiles the expression of 84 key genes central to the wound healing process and the genes are grouped into functions based the wound healing phases [9]. We selected one gene per group; *Col3a1* (extracellular matrix and cell adhesion), *Ccl12* (inflammatory cytokine and chemokines), *Tnf* (growth factors) and, *Wnt5a* (signal transduction).

References

1. Bryan, D.; Walker, K.B.; Ferguson, M.; Thorpe, R. Cytokine gene expression in a murine wound healing model. *Cytokine* **2005**, *31*, 429–438.
2. Feezor, R.J.; Paddock, H.N.; Baker, H.V.; Varela, J.C.; Barreda, J.; Moldawer, L.L.; Schultz, G.S.; Mozingo, D.W. Temporal patterns of gene expression in murine cutaneous burn wound healing. *Physiol Genomics* **2004**, *16*, 341–348.
3. Labus, M.B.; Stirk, C.M.; Thompson, W.D.; Melvin, W.T. Expression of wnt genes in early wound healing. *Wound Repair Regen* **1998**, *6*, 58–64.
4. Kagawa, S.; Matsuo, A.; Yagi, Y.; Ikematsu, K.; Tsuda, R.; Nakasono, I. The time-course analysis of gene expression during wound healing in mouse skin. *Leg. Med. (Tokyo)* **2009**, *11*, 70–75.
5. Benson, D.A.; Cavanaugh, M.; Clark, K.; Karsch-Mizrachi, I.; Lipman, D.J.; Ostell, J.; Sayers, E.W. Genbank. *Nucleic Acids Res.* **2017**, *45*, D37–D42.
6. Pundir, S.; Martin, M.J.; O'Donovan, C. Uniprot protein knowledgebase. *Methods Mol. Biol.* **2017**, *1558*, 41–55.

7. Kanehisa, M.; Furumichi, M.; Tanabe, M.; Sato, Y.; Morishima, K. Kegg: New perspectives on genomes, pathways, diseases and drugs. *Nucleic Acids Res.* **2017**, *45*, D353–D361.
8. Werner, S.; Grose, R. Regulation of wound healing by growth factors and cytokines. *Physiol Rev.* **2003**, *83*, 835–870.
9. Qiagen website. Mouse wound healing pcr array. http://www.sabiosciences.com/rt_pcr_product/HTML/PAMM-121A.html (accessed on 09 June 2017)
